# Chemotherapy-induced acetylation of ACLY by NAT10 promotes its nuclear accumulation and acetyl-CoA production to drive chemoresistance in hepatocellular carcinoma

**DOI:** 10.1038/s41419-024-06951-9

**Published:** 2024-07-31

**Authors:** Yuying Wang, Kunqi Su, Chang Wang, Tao Deng, Xiaofeng Liu, Shiqi Sun, Yang Jiang, Chunfeng Zhang, Baocai Xing, Xiaojuan Du

**Affiliations:** 1 Department of Cell Biology, School of Basic Medical Sciences, Peking University, Beijing, China; 2 Hepatopancreatobiliary Surgery Department I, Key Laboratory of Carcinogenesis and Translational Research (Ministry of Education/Beijing), Peking University Cancer Hospital & Institute, Beijing, China; 3 Department of Medical Genetics, School of Basic Medical Sciences, Peking University, Beijing, China

**Keywords:** Acetylation, Enzymes, Tumour biomarkers, Epigenetics

## Abstract

Chemotherapeutic efficacy is seriously impeded by chemoresistance in more than half of hepatocellular carcinoma (HCC) patients. However, the mechanisms involved in chemotherapy-induced upregulation of chemoresistant genes are not fully understood. Here, this study unravels a novel mechanism controlling nuclear acetyl-CoA production to activate the transcription of chemoresistant genes in HCC. NAT10 is upregulated in HCC tissues and its upregulation is correlated with poor prognosis of HCC patients. NAT10 is also upregulated in chemoresistant HCC cells. Targeting NAT10 increases the cytotoxicity of chemotherapy in HCC cells and mouse xenografts. Upon chemotherapy, NAT10 translocates from the nucleolus to the nucleus to activate the transcription of *CYP2C9* and *PIK3R1*. Additionally, nuclear acetyl-CoA is specifically upregulated by NAT10. Mechanistically, NAT10 binds with ACLY in the nucleus and acetylates ACLY at K468 to counteract the SQSTM1-mediated degradation upon chemotherapy. ACLY K468-Ac specifically accumulates in the nucleus and increases nuclear acetyl-CoA production to activate the transcription of *CYP2C9* and *PIK3R1* through enhancing H3K27ac. Importantly, K468 is required for nuclear localization of ACLY. Significantly, ACLY K468-Ac is upregulated in HCC tissues, and ablation of ACLY K468-Ac sensitizes HCC cells and mouse xenografts to chemotherapy. Collectively, these findings identify NAT10 as a novel chemoresistant driver and the blockage of NAT10-mediated ACLY K468-Ac possesses the potential to attenuate HCC chemoresistance.

## Introduction

Hepatocellular carcinoma (HCC) is the third leading cause of cancer death worldwide, with approximately 600,000 deaths per year [1]. Most HCC patients are diagnosed at advanced stages and lose the chance to undergo surgical resection [2]. Chemotherapy and tyrosine kinase inhibitors (TKIs) have been used as the major therapeutic approaches in advanced HCC patients [3]. Recently, it is reported that the chemotherapy-based hepatic arterial infusion (HAIC) avoids trans-arterial chemoembolization (TACE)-induced hypoxia, extends the overall survival of HCC patients by 5.1 months compared with sorafenib-based TKIs treatment and has become a promising therapeutic approach in the treatment of advanced HCC patients [4, 5]. However, about 54% of HCC patients have no response to chemotherapy-based HAIC due to chemoresistance [5]. Thus, a better understanding of the mechanisms underlying chemoresistance will provide novel strategies for improving the therapeutic efficacy in HCC.

Chemoresistance is brought about by the transcriptional upregulation of the genes related to drug transport, drug metabolism, cell survival signaling pathways and DNA damage repair [6–10]. Among these pathways, increased expression and activity of cytochrome P450 (P450) enzymes were found to decrease systemic drug levels by promoting drug metabolism in HCC [11]. More importantly, hyperactivation of the AKT signaling pathway promotes chemoresistance in HCC by inhibiting apoptosis and promoting cell survival [12]. Hence, the transcriptional upregulation of these genes plays driving roles in chemoresistance.

Gene transcription is activated by histone acetylation, which neutralizes histone-positive charges to loosen the interactions between histone and DNA, therefore facilitating the binding of transcription factors to the promoter [13–15]. Histone acetylation is highly sensitive to the availability of acetyl coenzyme A (acetyl-CoA), which is the obligatory acetyl donor for protein acetylation [16]. Given that acetyl-CoA is a high-energy and unstable molecule [16, 17], the rapid production of acetyl-CoA in the nucleus might ensure its availability for histone acetylation under DNA-damaging chemotherapy. In addition, the abundance of acetyl-CoA in distinct subcellular compartments is precisely controlled in response to physiological conditions or stresses [18]. Therefore, exploration of the mechanisms involved in the nuclear acetyl-CoA production will help better understand the upregulation of gene transcription in chemoresistance.

Acetyl-CoA is a central metabolic intermediate. The majority of acetyl-CoA is generated in the mitochondrial matrix, and powers the tricarboxylic acid (TCA) cycle and electron transport chain [18, 19]. Acetyl-CoA is also produced in the cytoplasm for several anabolic reactions [18, 19]. Recent study has found that acetyl-CoA is also produced in the nucleus in situ [20]. The enzymes responsible for the production of nuclear acetyl-CoA were also found in the nucleus, including pyruvate dehydrogenase complex (PDC), ATP citrate lyase (ACLY), and acyl-CoA synthetase short-chain family member 2 (ACSS2) [16, 18, 20]. Among these three acetyl-CoA production enzymes, only the nuclear ACLY level is upregulated and is responsible for the production of nuclear acetyl-CoA upon DNA damage [21–23]. However, how ACLY accumulates in the nucleus upon chemotherapy is not known.

N-acetyltransferase 10 (NAT10, also named as ALP and Kre33) was initially discovered as a protein acetyltransferase acetylating the upstream binding factor (UBF) to activate RNA polymerase I transcription [24]. Later, studies found that NAT10 plays as a cytidine acetyltransferase, which acetylates 18S rRNA to promote pre-rRNA processing and acetylates tRNA for correct translation [25, 26]. NAT10 also acetylates mRNA to promote its stability and translation efficiency [27]. Additionally, NAT10 participates in multiple cellular biological processes via its acetyltransferase activity including mitosis, meiosis, and senescence [28–34]. Moreover, NAT10 plays an important role in response to cellular stresses. Upon energy stress, deacetylation of NAT10 promotes the transition from rRNA biogenesis to autophagy [35]. Under DNA damage, NAT10 acetylates p53 and MORC2 to control cell-cycle checkpoint and acetylates PARP1 to promote DNA damage repair [36–38]. Therefore, NAT10 is upregulated and plays essential roles in the progression of various cancers including pancreatic cancer, gastric cancer, and colorectal cancer [39, 40]. NAT10 is upregulated in HCC and is associated with poor survival of the patients [41]. Additionally, NAT10 promotes ER stress-mediated metastasis and lenvatinib resistance in HCC through mediating ac4C-modified HSP90AA1 RNA acetylation [42]. It is also reported that NAT10 enhances doxorubicin resistance in HCC with unknown mechanism [43]. However, if NAT10 controls gene transcription in HCC chemoresistance remains unclear.

In the present study, we identify NAT10 as a driver of chemoresistance in HCC cells and mouse xenografts in vivo. We used mass spectrometry and transcriptome sequencing to elucidate the molecular mechanisms underlying the role of NAT10 in chemoresistance. We demonstrate that ACLY is a key substrate of NAT10 and the acetylation at lysine 468 is critical for NAT10-mediated chemoresistance. Furthermore, ACLY K468-Ac increases the nuclear acetyl-CoA production and activates *CYP2C9* and *PIK3R1* transcription through enhancing H3K27 acetylation on the promoter. Loss of ACLY K468 acetylation significantly alleviates chemoresistance in HCC cells and mouse xenografts in vivo. Importantly, K468 is required for the nuclear localization of ACLY. Thus, targeting NAT10-mediated ACLY acetylation at K468 provides a promising strategy for alleviating HCC chemoresistance.

## Results

### Targeting NAT10 sensitizes HCC cells and mouse xenografts to chemotherapy

**Fig. 1:**
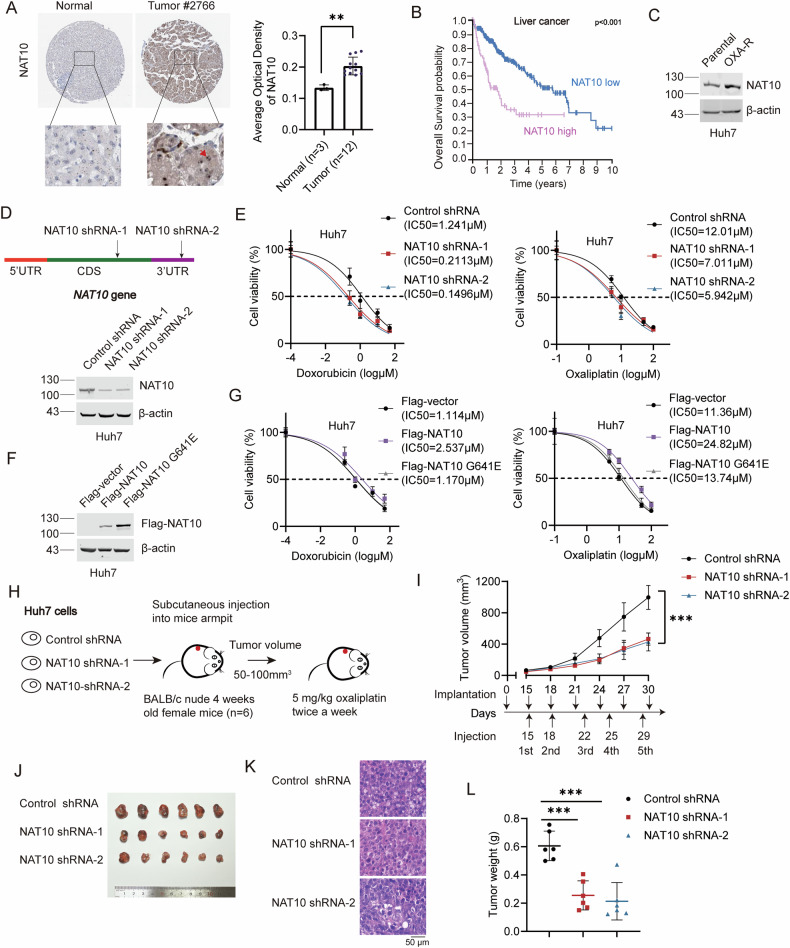
Targeting NAT10 sensitizes HCC cells and mouse xenografts to chemotherapy. **A** IHC staining of NAT10 in HCC and normal liver tissues from the HPA database. Average optical density data were analyzed by one-way ANOVA and presented as mean ± SEM **P < 0.01. **B** Kaplan–Meier survival analysis of the overall survival probability of patients with HCC according to NAT10 expression (high, *n* = 79; low, *n* = 286). **C** Western blot (WB) analysis of NAT10 expression in OXA-resistant Huh7 cells and parental Huh7 cells. **D** The schematic diagram of NAT10’s shRNAs (upper panel). WB was performed on the cell lysates to evaluate NAT10 levels in Huh7-NAT10 shRNA-1, Huh7-NAT10 shRNA-2, and Huh7-control shRNA cells (lower panel). **E** The IC50 of doxorubicin and oxaliplatin in Huh7-NAT10 shRNA-1, Huh7-NAT10 shRNA-2, and Huh7-control shRNA cells. Data were analyzed by one-way ANOVA and presented as mean ± SD (*n* = 3). **F** Huh7 cells were transfected with Flag-vector, Flag-NAT10 or Flag-NAT10 G641E plasmids. WB was performed on the cell lysates to evaluate Flag-NAT10 and Flag-NAT10 G641E levels. **G** The IC50 of doxorubicin and oxaliplatin in Huh7 cells expressing Flag, Flag-NAT10 or Flag-NAT10 G641E. Data were analyzed by one-way ANOVA and presented as mean ± SD (*n* = 3). **H** Huh7-NAT10 shRNA-1, Huh7-NAT10 shRNA-2, and Huh7-control shRNA cells were subcutaneously implanted into nude mice. Oxaliplatin (5 mg/kg) was intraperitoneally injected twice a week after Huh7 cells injection (*n* = 6). **I** The volumes of tumor xenografts were shown. Data were analyzed by one-way ANOVA and presented as mean ± SD, ***P < 0.001. **J** Tumors were dissected at the end of the experiment. **K** Tumor tissues were stained with HE. Scale bar, 50 μm. **L** The weights of tumor xenografts were shown. Data were analyzed by one-way ANOVA and presented as mean ± SD, ***P < 0.001.

To evaluate the expression level of NAT10 in human HCC tissues, IHC data from the Human Protein Atlas (HPA) database was analyzed. NAT10 was upregulated in HCC tumor tissues compared with non-tumorous liver tissues (Fig. 1A). Furthermore, high expression level of NAT10 was associated with shorter survival (Fig. 1B), indicating that targeting NAT10 might improve the outcome of HCC patients. To explore the function of NAT10 in the chemoresistance in HCC, we generated oxaliplatin-resistant Huh7 (Huh7-OXA-R) cells and found that NAT10 expression was upregulated in Huh7-OXA-R cells compared with the parental cells (Fig. 1C). To further confirm the function of NAT10 in the chemoresistance in HCC, we evaluated the IC50 of doxorubicin and oxaliplatin in Huh7-NAT10 shRNA-1, Huh7-NAT10 shRNA-2 or Huh7-control shRNA cells. Knockdown of NAT10 reduced the IC50 of these chemotherapeutic agents (Fig. 1D, E). In addition, the knockdown of NAT10 inhibits HCC cell growth and induces apoptosis under OXA and doxorubicin (DOX) treatment (Fig. S1A, B). These results indicate that targeting NAT10 sensitizes HCC cells to chemotherapy. Additionally, ectopic Flag-NAT10 enhanced chemoresistance in HCC cells, while the acetyltransferase enzyme-dead mutant Flag-NAT10 G641E failed to do so (Fig. 1F, G). These results indicate that NAT10 promotes HCC chemoresistance depending on its acetyltransferase activity.

To further confirm the function of NAT10 in chemoresistance in vivo, we subcutaneously implanted Huh7-NAT10 shRNA-1, Huh7-NAT10 shRNA-2, and Huh7-control shRNA cells into the BALB/c nude mice. When the tumor volume reached 50–100 mm^3^, oxaliplatin was injected intraperitoneally twice a week, and the experiment was ended when the maximum diameter of tumors reached 1.5 cm (Fig. 1H). Knockdown of NAT10 significantly reduced the tumor volume and weight in the mouse xenograft model (Fig. 1I–L). Thus, we demonstrate that NAT10 promotes chemoresistance in HCC cells and in vivo.

### Chemotherapy induces NAT10-mediated transcriptional activation of *CYP2C9* and *PIK3R1*

**Fig. 2:**
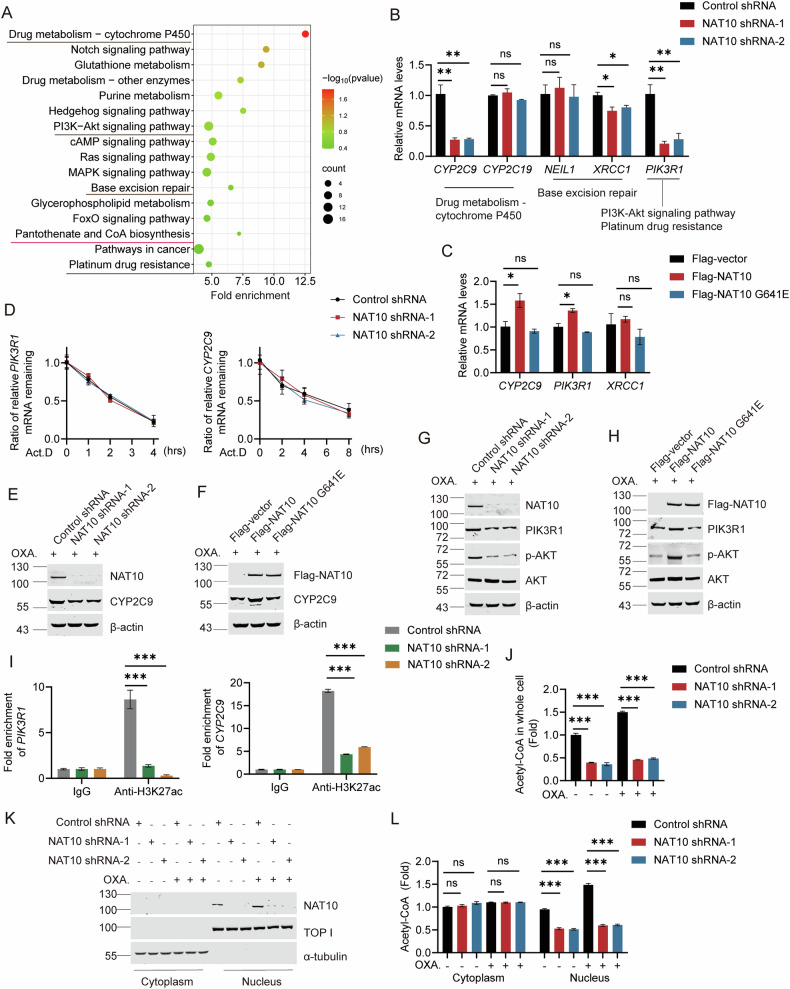
NAT10 controls nuclear acetyl-CoA production to activate the transcription of chemotherapy-resistant genes. **A** The enriched pathways of the NAT10 downregulated genes were plotted by KEGG enrichment analysis using the data obtained by RNA-seq of the NAT10 shRNA and control cells after doxorubicin treatment. **B** The mRNA levels of *CYP2C9, CYP2C19, NEIL1, XRCC1,* and *PIK3R1* genes were evaluated by RT-qPCR in Huh7-NAT10 shRNA-1, Huh7-NAT10 shRNA-2 and Huh7-control shRNA cells after doxorubicin treatment. Data were analyzed by one-way ANOVA and presented as mean ± SEM (*n* = 3), ns denotes no significance, *P < 0.05, **P < 0.01. **C** Huh7 cells were transfected with Flag-vector, Flag-NAT10 or Flag-NAT10 G641E plasmids and treated with doxorubicin. The mRNA levels of *CYP2C9*, *XRCC1* and *PIK3R1* genes were evaluated by RT-qPCR. Data were analyzed by one-way ANOVA and presented as mean ± SEM (*n* = 3), ns denotes no significance, *P < 0.05. **D** Huh7-NAT10 shRNA-1, Huh7-NAT10 shRNA-2, and Huh7-control shRNA cells were treated with oxaliplatin and then were treated with actinomycin D (Act.D). These cells were collected at different time points for RNA extraction. The mRNA levels of *PIK3R1* and *CYP2C9* genes were detected by RT-qPCR. **E**, **G** The protein levels of PIK3R1, p-AKT, AKT, and CYP2C9 were detected by WB in Huh7-NAT10 shRNA-1, Huh7-NAT10 shRNA-2 and Huh7-control shRNA cells with oxaliplatin treatment. **F**, **H** Huh7 cells were transfected with Flag-vector, Flag-NAT10 or Flag-NAT10 G641E plasmids and treated with oxaliplatin. The protein levels of PIK3R1, p-AKT, AKT, and CYP2C9 were detected by WB. **I** The H3K27ac levels on the promoters of *PIK3R1* and *CYP2C9* genes were detected by ChIP in Huh7-NAT10 shRNA-1, Huh7-NAT10 shRNA-2, and Huh7-control shRNA cells with 10 μM oxaliplatin treatment. Data were analyzed by one-way ANOVA and presented as mean ± SEM (*n* = 3), ***P < 0.001. **J** The acetyl-CoA levels were analyzed in Huh7-NAT10 shRNA-1, Huh7-NAT10 shRNA-2, and Huh7-control shRNA cells with or without oxaliplatin treatment. Data were analyzed by one-way ANOVA and presented as mean ± SEM (*n* = 3), ***P < 0.001. **K** Cellular fraction was prepared in Huh7-NAT10 shRNA-1, Huh7-NAT10 shRNA-2, and Huh7-control shRNA cells with or without oxaliplatin treatment. **L** The acetyl-CoA levels were analyzed in **K**’s cellular fractions. Data were analyzed by one-way ANOVA and presented as mean ± SEM (*n* = 3), ns denotes no significance, ***P < 0.001.

To uncover the mechanism by which NAT10 promotes chemoresistance, transcriptome profile was obtained from NAT10 knockdown and control cells after chemotherapy treatment. The downregulated transcripts were analyzed by KEGG, which shows that the transcripts were enriched in the drug metabolism, PI3K-AKT signaling pathway, base excision repair, platinum drug resistance and other drug resistance-related pathways (Fig. 2A). Interestingly, the downstream genes were also enriched in the pantothenate and CoA biosynthesis, suggesting that NAT10 might control the metabolism of pantothenate and CoA, in addition to the transcriptional regulation of the chemotherapy-resistant genes.

Next, we evaluated the expression of the key genes in the enriched pathways controlled by NAT10. The RT-qPCR results showed that the mRNA levels of *CYP2C9*, *XRCC1,* and *PIK3R1* genes were significantly reduced in NAT10-knockdown cells (Fig. 2B). Correspondingly, the mRNA levels of *CYP2C9* and *PIK3R1* genes were upregulated by ectopic Flag-NAT10, but not Flag-NAT10 G641E (Fig. 2C), suggesting that NAT10 activates the transcription of *CYP2C9* and *PIK3R1* genes depending on its acetyltransferase activity. It is known that NAT10 possesses the capability of acetylating mRNA to regulate the stability of the mRNA [44]. To determine if NAT10 regulates the mRNA stability of the downstream genes upon chemotherapy, we evaluated the half-life of *PIK3R1* and *CYP2C9* mRNA. The mRNA stability of *PIK3R1* and *CYP2C9* was not changed in NAT10-knockdown cells (Fig. 2D), indicating that NAT10 drives HCC chemoresistance through activating the transcription of *PIK3R1* and *CYP2C9* rather than regulating the mRNA stability of these genes.

We further verified that NAT10 upregulates the protein levels of CYP2C9, which acts in the drug metabolism, and PIK3R1 to activate the PI3K-AKT pathway (phosphorylation of AKT) depending on its acetyltransferase activity (Fig. 2E–H and Fig. S2A–D). These findings demonstrate that NAT10 activates the transcription of the genes controlling drug metabolism and the PI3K-AKT pathway to drive HCC chemoresistance.

### NAT10 promotes H3K27 acetylation of downstream genes on the promoters and the production of nuclear acetyl-CoA upon chemotherapy

Since the gene transcription of *PIK3R1* and *CYP2C9* is activated by H3K27 acetylation (H3K27ac) on the promoter [45, 46], the H3K27ac levels on *PIK3R1* and *CYP2C9* promoters were evaluated by chromatin precipitation (ChIP) experiment. The H3K27ac levels on *PIK3R1* and *CYP2C9* promoters were reduced in NAT10-knockdown cells (Fig. 2I), indicating that NAT10 activates the transcription of chemotherapy-resistant genes by enhancing H3K27ac.

The RNA-seq showed that downstream genes of NAT10 were enriched in the CoA synthesis pathway under chemotherapeutic reagent’s treatment. Therefore, we further determined if acetyl-CoA level is controlled by NAT10. Depletion or knockdown of NAT10 significantly reduced the acetyl-CoA level (Fig. 2J and Fig. S3A). We further showed that depletion or knockdown of NAT10 significantly reduced the nuclear acetyl-CoA level rather than the cytoplasmic acetyl-CoA level (Fig. 2K, L and Fig. S3B, C). These results suggest that NAT10 increases the production of nuclear acetyl-CoA to epigenetically activate the transcription of the downstream genes.

### Chemotherapy induces the binding of NAT10 with ACLY in the nucleus

**Fig. 3:**
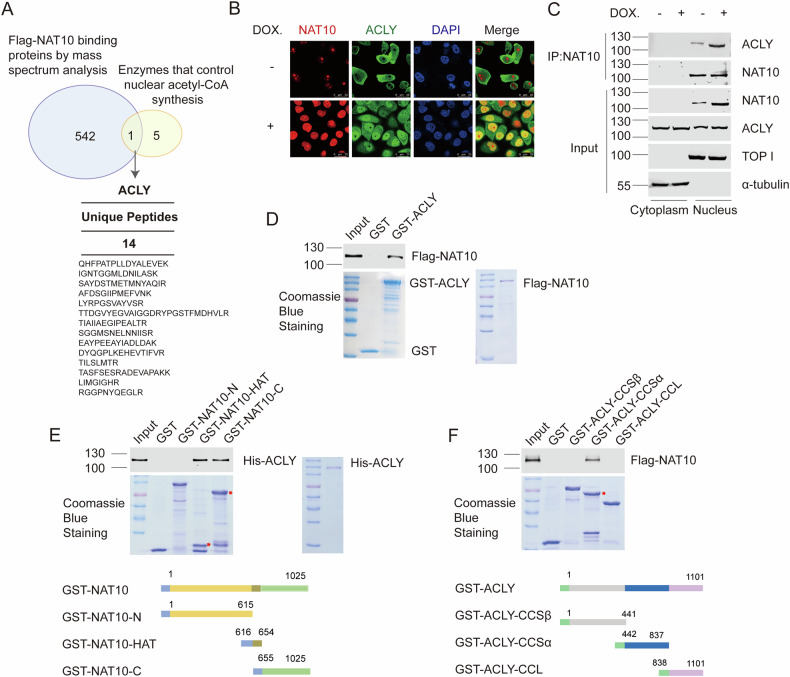
Chemotherapy induces the binding of NAT10 with ACLY in the nucleus. **A** Venn diagram showed the common proteins between the nuclear proteins responsible for the production of acetyl-CoA including ACLY, ACSS2, pyruvate dehydrogenase (E1), dihydrolipoamide transacetylase(E2), dihydrolipoamide dehydrogenase (E3), as well as the tethering protein, E3-binding protein (E3BP) and the Flag-NAT10-binding proteins in HeLa cells [28]. The blue area illustrates the number of Flag-NAT10-binding proteins, the yellow area illustrates the number of nuclear proteins responsible for the production of acetyl-CoA, and the gray area illustrates the number of common proteins between two datasets. **B** Immunofluorescent staining showed the colocalization of ACLY (green) and NAT10 (red) treated with doxorubicin. Scale bar, 25 μm. **C** Cells were treated with doxorubicin. The cytoplasmic lysate and nuclear extraction were fractioned, which were subjected to immunoprecipitation using anti-NAT10 antibody. The immunoprecipitates were subsequently immunoblotted with the indicated antibodies. Topoisomerase I (TOP I) and α-tubulin were used as nuclear and cytoplasmic marker, respectively. **D** GST pull-down was performed with purified Flag-NAT10 and GST-ACLY proteins. ACLY bound Flag-NAT10 was evaluated by WB using anti-Flag antibody. The amounts of GST fusion proteins and purified Flag-NAT10 protein used in the GST pull-down were shown by Coomassie blue staining. **E** GST pull-down was performed with purified His-ACLY and GST-NAT10’s deletion mutants. The amounts of GST fusion proteins and purified Flag-NAT10 protein used in the GST pull-down experiments were shown by Coomassie blue staining. NAT10-bound His-ACLY was evaluated by WB using the anti-His antibody. The schematic diagram represents the GST-NAT10 deletion mutant constructs [28] (lower panel). **F** GST pull-down was performed with purified Flag-NAT10 and GST-ACLY’s deletion mutants. The amounts of GST fusion proteins used in the GST pull-down were shown by Coomassie blue staining. ACLY bound Flag-NAT10 was evaluated by WB using the anti-Flag antibody. The schematic diagram represents the GST-ACLY deletion mutant constructs [64] (lower panel).

To uncover the mechanism by which NAT10 controls the nuclear acetyl-CoA level, we looked for the enzymes involved in the nuclear acetyl-CoA production in the NAT10-binding proteins by Wayne analysis. We found that ACLY, a key enzyme that produces acetyl-CoA in the nucleus, was among the NAT10-binding proteins (Fig. 3A). To obtain functional insight into the association between NAT10 and ACLY, we examined the cellular localization of NAT10 and ACLY upon chemotherapy. NAT10 translocated from the nucleolus to the nucleus, and co-localized with ACLY under doxorubicin treatment (Fig. 3B). The drug-induced binding between NAT10 and ACLY was confirmed by co-immunoprecipitation (Fig. 3C). These results indicate that NAT10 might regulate ACLY function in the nucleus upon DNA-damaging chemotherapy.

To determine if NAT10 directly interacts with ACLY, GST pull-down experiment was performed with purified Flag-NAT10 protein from *sf9* cells and GST-ACLY protein from *E. coli*. Purified Flag-NAT10 directly bound GST-ACLY in vitro (Fig. 3D). To map the ACLY-binding domain in NAT10, GST pull-down experiments were performed with the purified GST-NAT10 deletion mutants and His-ACLY protein. ACLY bound to NAT10-C-terminus and NAT10-HAT (Fig. 3E), which contains the acetyltransferase activity domain, suggesting that NAT10 might regulate ACLY function through its acetyltransferase function. To further explore the minimal binding domain in ACLY, we constructed plasmids expressing truncated GST-ACLY and purified the corresponding fusion proteins. GST-ACLY bound NAT10 with the CCSα domain (Fig. 3F). These results demonstrate that NAT10 directly binds ACLY in the nucleus upon DNA-damaging chemotherapy.

### NAT10 controls ACLY protein level depending on its acetyltransferase activity

**Fig. 4:**
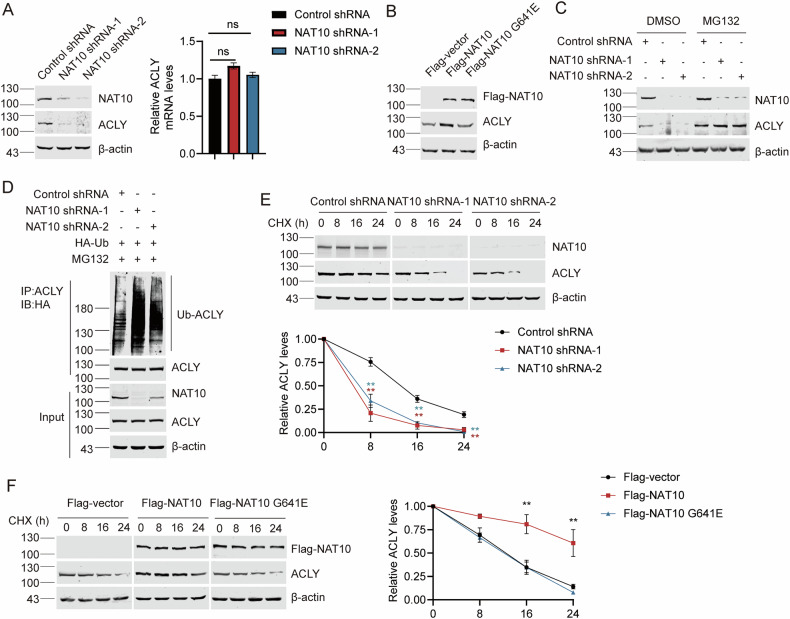
NAT10 controls ACLY protein level depending on its acetyltransferase activity. **A** WB was performed on the cell lysates to evaluate NAT10 and ACLY protein levels in Huh7-NAT10 shRNA-1, Huh7-NAT10 shRNA-2, and Huh7-control shRNA cells. ACLY mRNA level was evaluated by RT-qPCR. Data were analyzed by one-way ANOVA and presented as mean ± SEM (*n* = 3), ns denotes no significance. **B** Huh7 cells were transfected with Flag-vector, Flag-NAT10 or Flag-NAT10 G641E plasmids. WB was performed on the cell lysates to evaluate ACLY levels. **C** Huh7-NAT10 shRNA-1, Huh7-NAT10 shRNA-2, and Huh7-control shRNA cells were treated with 10 μM MG132, and ACLY levels were detected by WB. **D** Huh7-NAT10 shRNA-1, Huh7-NAT10 shRNA-2, and Huh7-control shRNA cells were treated with 10 μM MG132 and transfected with HA-Ub plasmid. Then lysates were subjected to immunoprecipitation using anti-ACLY antibody. The ubiquitination levels of ACLY were evaluated by WB using the anti-HA antibody. **E** Huh7-NAT10 shRNA-1, Huh7-NAT10 shRNA-2, and Huh7-control shRNA cells were treated with cycloheximide (CHX), and harvested at the indicated time points. Proteins from cell lysates were subjected to immunoblot for the evaluation of NAT10 and ACLY (left panel). Relative ACLY protein levels at indicated time points were plotted (right panel). Data were analyzed by *T*-test and presented as mean ± SEM (*n* = 3), *P < 0.05, **P < 0.01. **F** Huh7 cells were transfected with Flag-vector, Flag-NAT10 or Flag-NAT10 G641E plasmids and harvested at indicated time points after treatment with CHX. Proteins from cell lysates were immunoblotted with the antibodies as indicated (left panel). Relative ACLY protein levels at indicated time points were shown (right panel). Data were analyzed by one-way ANOVA and presented as mean ± SEM (*n* = 3), **P < 0.01.

To determine if and how NAT10 regulates ACLY function, we evaluated the ACLY level in Huh7-NAT10-shRNA-1, Huh7-NAT10-shRNA-2, and Huh7-control-shRNA cells. Results showed that ACLY protein levels decreased in Huh7-NAT10-shRNA-1, Huh7-NAT10-shRNA-2 compared with Huh7-control-shRNA cells, without affecting ACLY mRNA level (Fig. 4A). Additionally, knockdown of NAT10 by sgRNA or siRNA in HeLa cells also leads to the decease of ACLY (Fig. S4A, B), indicating that NAT10 upregulates the ACLY level through protein-protein interaction. In contrast, ectopic Flag-NAT10 increased ACLY level, but Flag-NAT10 G641E failed to do so (Fig. 4B), indicating that NAT10 upregulates ACLY level depending on its acetyltransferase activity.

We thereafter explored if NAT10 regulates ACLY level through proteasome mediated pathway. NAT10 knockdown-induced decrease of ACLY was blocked by MG132, a proteasome inhibitor (Fig. 4C). Further, the knockdown of NAT10 enhanced poly-ubiquitination of ACLY (Fig. 4D), demonstrating that NAT10 protects ACLY from the ubiquitination-proteasome mediated degradation. The half-life of ACLY was significantly reduced in the NAT10-knockdown cells (Fig. 4E). In contrast, ectopic Flag-NAT10 instead of Flag-NAT10 G641E prolonged the half-life of ACLY (Fig. 4F). Taken together, our data indicate that NAT10 stabilizes ACLY depending on its acetyltransferase activity.

### Chemotherapy induces the nuclear translocation of NAT10 to control the nuclear ACLY level

**Fig. 5:**
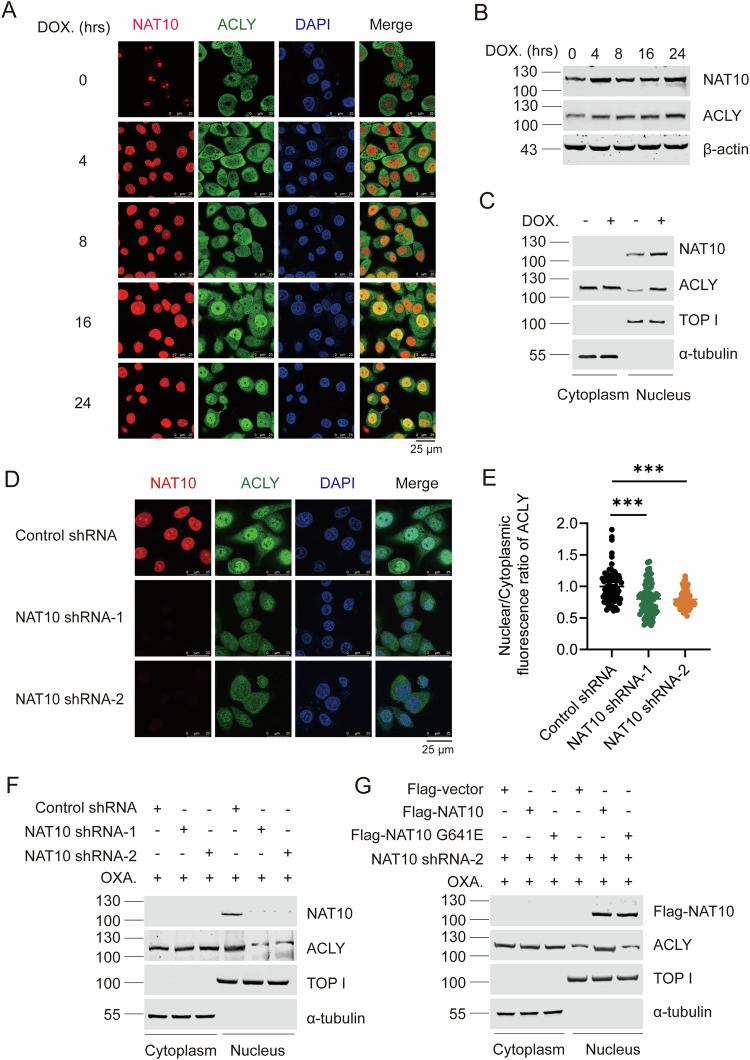
Chemotherapy induces the nuclear translocation of NAT10 to control the nuclear ACLY level. **A** Immunofluorescent staining showed the colocalization of ACLY (green) and NAT10 (red) treated for 0, 4, 8, 16, or 24 h with 5 μM doxorubicin. Scale bar, 25 μm. **B** Then the protein levels of NAT10 and ACLY were analyzed by WB. **C** Cells were treated with or without doxorubicin. The cytoplasmic lysate and nuclear extraction were fractioned, which were subjected to WB using indicated antibodies. TOP I and α-tubulin were used as nuclear and cytoplasmic marker, respectively. **D** Immunofluorescent staining showed the fluorescent intensity of ACLY (green) and NAT10 (red) in NAT10 shRNA-1, NAT10 shRNA-2, and control shRNA cells treated with 5 μM doxorubicin. Scale bar, 25 μm. **E** The fluorescent intensity of ACLY was determined by scanning with Leica LAS X confocal software (*n* = 60~80 cells). The relative nuclear/cytoplasmic fluorescent intensity standardized by that in the control cells was assigned as 100%. Data were analyzed by *T*-test and presented as mean ± SEM, **P < 0.01. **F** Huh7-NAT10 shRNA-1, Huh7-NAT10 shRNA-2, and Huh7-control shRNA cells were treated with 10 μM oxaliplatin. The cytoplasmic lysate and nuclear extraction were fractioned, which were subjected to WB using indicated antibodies. TOP I and α-tubulin were used as nuclear and cytoplasmic marker, respectively. **G** Huh7-NAT10 shRNA-2 cells were transfected with Flag-vector, Flag-NAT10 G641E, Flag-NAT10 plasmids and treated with 10 μM oxaliplatin. The cytoplasmic lysate and nuclear extraction were fractioned, which were subjected to WB using indicated antibodies. TOP I and α-tubulin were used as nuclear and cytoplasmic marker, respectively.

To unravel the regulatory effect of NAT10 on ACLY upon DNA-damaging chemotherapy, we examined the expression and localization of NAT10 and ACLY under doxorubicin treatment. Immunofluorescent staining results showed that NAT10 translocated from the nucleolus to the nucleoplasm ahead of the nuclear accumulation of ACLY upon chemotherapy (Fig. 5A). It is notable that ACLY and NAT10 gradually increased along with the extension of the doxorubicin treatment duration (Fig. 5B). We further confirmed that NAT10 and the nuclear ACLY levels are upregulated while the cytoplasmic ACLY level is not changed (Fig. 5C). These results indicated that NAT10 might control the nuclear level of ACLY upon DNA-damaging chemotherapy. Further, immunofluorescent staining showed that the nuclear ACLY level was significantly reduced in NAT10-knockdown cells under DNA damage chemotherapeutic drug treatment (Fig. 5D, E), which was confirmed by Western blot (Fig. 5F and Fig. S5A). Ectopic Flag-NAT10 rescued NAT10 knockdown-mediated decrease of the nuclear ACLY level, but Flag-NAT10 G641E lost this capability (Fig. 5G and Fig. S5B). These results demonstrate that NAT10 controls nuclear ACLY level depending on its acetyltransferase activity upon DNA-damaging chemotherapy.

### NAT10 acetylates ACLY at lysine 468 to stabilize ACLY

**Fig. 6:**
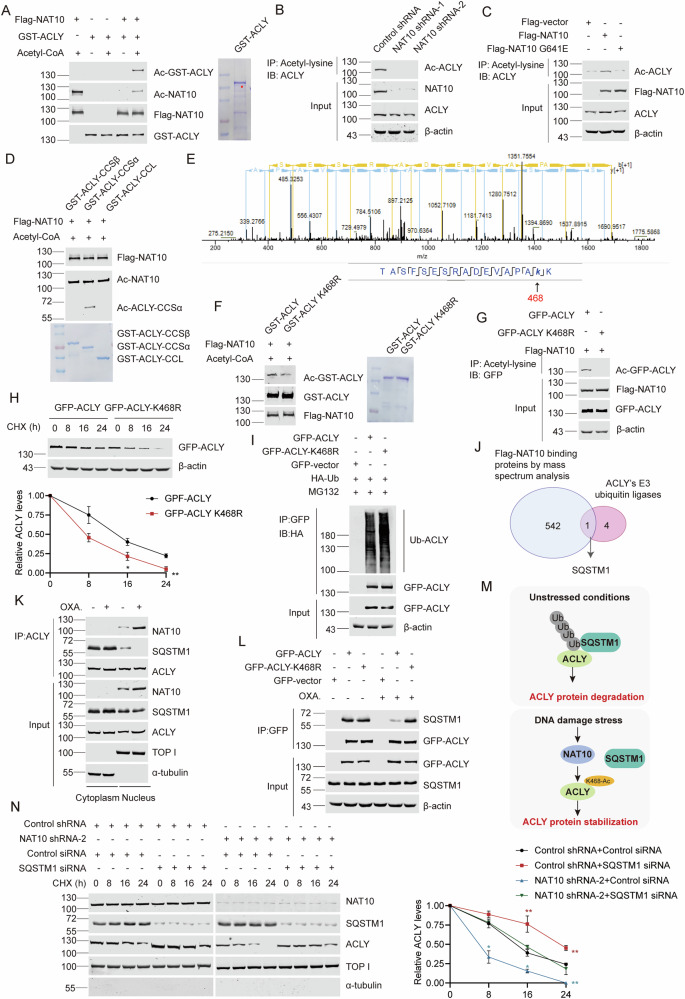
NAT10 acetylates ACLY at lysine 468 to stabilize ACLY. **A** In vitro acetylation was performed with purified GST-ACLY and Flag-NAT10 proteins with or without acetyl-CoA. The reaction products were detected by WB. The purified GST-ACLY used in the experiment was shown by Coomassie blue staining. **B** Huh7-NAT10 shRNA-1, Huh7-NAT10 shRNA-2, and Huh7-control shRNA cells were subjected to immunoprecipitation using anti-acetyl-lysine. The acetylation levels of ACLY were evaluated by WB using anti-ACLY. **C** Cells were transfected with indicated plasmids. Proteins were immunoprecipitated using anti-acetyl-lysine and the acetylation levels of ACLY were evaluated by WB using anti-ACLY. **D** In vitro acetylation experiment was performed with indicated proteins and acetyl-CoA. The acetylation levels of GST-ACLY truncated proteins were evaluated by WB using anti-acetyl-lysine. The amounts of GST fusion proteins used in the experiments were shown by Coomassie blue staining. **E** In vitro acetylation experiment was performed as (**A**) and the reaction products were resolved by SDS-PAGE. The band of acetylated ACLY was cut, fully trypsinized, and analyzed by LC–MS/MS (Thermo). Mass spectrometry data were processed using the Proteome Discoverer software (Version 1.4). **F** In vitro acetylation experiment was performed with indicated proteins and acetyl-CoA. The acetylation levels of GST-ACLY mutant proteins were evaluated by WB using anti-acetyl-lysine. The amounts of GST fusion proteins used in the experiments were shown by Coomassie blue staining. **G** Cells were transfected with indicated plasmids. Proteins were immunoprecipitated using anti-acetyl-lysine and the acetylation levels of ACLY were evaluated by WB using anti-GFP. **H** Cells were transfected with indicated plasmids and treated with CHX at indicated time points. Proteins from cell lysates were immunoblotted with the antibodies as indicated (upper panel). Relative GFP-ACLY protein levels at different time points were shown (lower panel). Data were analyzed by one-way ANOVA and presented as mean ± SEM (*n* = 3), *P < 0.05, **P < 0.01. **I** Cells were transfected with indicated plasmids and treated with 10 μM MG132. Then lysates were subjected to immunoprecipitation using anti-GFP. The ubiquitination levels of ACLY were evaluated by WB using anti-HA. **J** Venn diagram showed the common proteins between the ACLY’s E3 ubiquitin ligase including UBR4, Cullin3 (CUL3), Neuronally expressed developmentally down-regulated 4 (NEDD4), SQSTM1, Hrd1, and the Flag-NAT10-binding proteins in HeLa cells [28]. The blue area illustrates the number of Flag-NAT10-binding proteins, the red area illustrates the number of ACLY’s E3 ubiquitin ligase, and the gray area illustrates the number of common proteins between two datasets. **K** Huh7 cells were treated with 10 μM oxaliplatin. The cytoplasmic lysate and nuclear extraction were fractioned, which were subjected to immunoprecipitation using anti-ACLY antibody. The immunoprecipitates were subsequently immunoblotted with the indicated antibodies. TOP I and α-tubulin were used as nuclear and cytoplasmic marker, respectively. **L** Huh7 cells were transfected with indicated plasmids. Proteins were immunoprecipitated using anti-GFP. The immunoprecipitates were subsequently immunoblotted with the indicated antibodies. **M** Schematic model of ACLY protein level regulated by SQSTM1 under unstressed or DNA damage stress conditions. ACLY binds to SQSTM1 and is degraded by SQSTM1-mediated ubiquitination under unstressed condition. However, ACLY is acetylated by NAT10 after DNA damage which prevents the binding between ACLY and SQSTM1, thus leading to increased ACLY protein level. **N** Huh7-control shRNA and Huh7-NAT10 shRNA-2 cells were transfected with control siRNA or SQSTM1 siRNA and then harvested at indicated time points after treatment with CHX. Proteins from cell lysates were immunoblotted with the antibodies as indicated (left panel). Relative ACLY protein levels at indicated time points were shown (right panel). Data were analyzed by one-way ANOVA and presented as mean ± SEM (*n* = 3), *P < 0.05, **P < 0.01.

To explore if NAT10 acetylates ACLY, we performed in vitro acetylation experiment with purified Flag-NAT10 and GST-ACLY proteins. GST-ACLY was acetylated in the presence of both acetyl-CoA and Flag-NAT10 (Fig. 6A). In addition, the acetylation level of ACLY decreased in Huh7-NAT10-shRNA-1, Huh7-NAT10-shRNA-2 and HeLa-NAT10 sgRNA cells (Fig. 6B and Fig. S6). Ectopic Flag-NAT10 increased the acetylation level of ACLY, while Flag-NAT10 G641E lost this function (Fig. 6C). These data demonstrate that NAT10 is an acetyltransferase of ACLY. To further narrow down the domain containing the acetylation site in ACLY, in vitro acetylation experiment was performed with truncated GST-ACLY fusion proteins and Flag-NAT10 protein. NAT10 acetylated the ACLY CCSα domain (Fig. 6D), which directly bound to NAT10.

Next, we determined the acetylation sites of ACLY by mass spectrometric analysis after in vitro acetylation experiment performed with purified GST-ACLY and Flag-NAT10 proteins. ACLY was acetylated by NAT10 at lysine 468 (Fig. 6E). K468 site was found to be conserved among multiple species (Fig. S7), suggesting that K468 acetylation might be required for ACLY function. To further verify the reliability of K468 acetylation, GST-ACLY K468R (loss of-acetylation mutant) was purified from *E. coli* bacterial cells. In vitro acetylation experiments showed that the acetylation level of GST-ACLY K468R significantly decreased (Fig. 6F). In addition, GFP-ACLY K468R was not able to be acetylated by Flag-NAT10 in cells (Fig. 6G). These results confirm that NAT10 acetylates ACLY at K468.

We next evaluated the protein stability of GFP-ACLY K468R. The half-life of GFP-ACLY K468R was significantly reduced compared with GFP-ACLY (Fig. 6H). Additionally, the poly-ubiquitination level of GFP-ACLY K468R significantly increased (Fig. 6I), suggesting that the acetylation of ACLY K468 stabilizes ACLY through blocking the ubiquitin-proteasomal degradation. To further explore the mechanism by which NAT10-mediated ACLY K468 acetylation affects the poly-ubiquitination level of ACLY, we screened the E3 ubiquitin ligases for ACLY in the NAT10-binding proteins by Wayne analysis. SQSTM1, an E3 ubiquitin ligase for ACLY, was among the NAT10-binding proteins (Fig. 6J). We found that NAT10 competed with SQSTM1 to bind ACLY in the nucleus upon doxorubicin and oxaliplatin treatment (Fig. 6K and Fig. S8A). Furthermore, the loss of ACLY K468 acetylation enhanced the binding between ACLY and SQSTM1 (Fig. 6L and Fig. S8B). NAT10-mediated stabilization of ACLY in the nucleus is dependent on SQSTM1 (Fig. 6N). These data demonstrate that NAT10-mediated K468 acetylation abrogates SQSTM1-mediated nuclear ACLY degradation to increase the nuclear ACLY level upon DNA-damaging chemotherapy (Fig. 6M).

### ACLY K468-Ac occurs upon chemotherapy and increases the production of nuclear acetyl-CoA to activate the transcription of chemotherapy-resistant genes

**Fig. 7:**
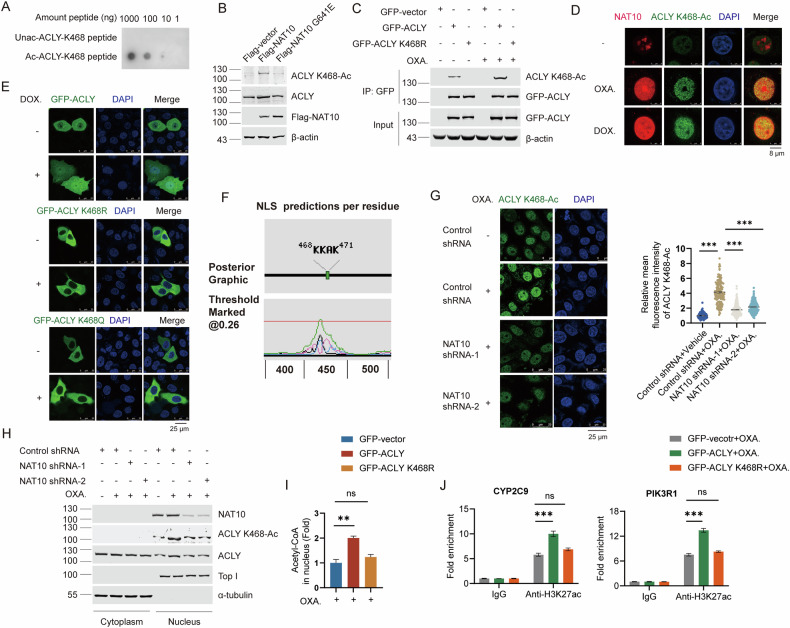
ACLY K468-Ac occurs upon chemotherapy and increases the production of nuclear acetyl-CoA to activate the transcription of chemotherapy-resistant genes. **A** Dot blotting was performed with unacetyl-ACLY peptide or acetyl-ACLY K468 peptide to detect the specificity of ACLY K468-Ac antibody. **B** Huh7 cells were transfected with Flag-vector, Flag-NAT10, Flag-NAT10 G641E plasmids. The acetylation levels of ACLY were evaluated by WB using anti-ACLY K468-Ac. **C** Huh7 cells were transfected with GFP-vector, GFP-ACLY, GFP-ACLY K468R plasmids and treated with oxaliplatin. Co-immunoprecipitation was performed with anti-GFP and the acetylation levels of GFP-ACLY was evaluated by WB using anti-ACLY K468-Ac. **D** Immunofluorescent staining showed the colocalization of ACLY K468-Ac (green) and NAT10 (red) in cells treated with oxaliplatin or doxorubicin. Scale bar, 8 μm. **E** Immunofluorescent staining showed the localization of GFP-ACLY, GFP-ACLY K468R, and GFP-ACLY K468Q treated with or without doxorubicin. Scale bar, 25 μm. **F** The nuclear localization signal sequence of ACLY was analyzed by NLStradamus. **G** Immunofluorescent staining showed the fluorescent intensity of ACLY K468-Ac (green) in NAT10 shRNA-1, NAT10 shRNA-2, and control shRNA cells treated with oxaliplatin. Scale bar, 25 μm (left panel). The fluorescent intensity of ACLY K468-Ac was determined by scanning with Leica LAS X confocal software (*n* = 100~120 cells). The relative fluorescent intensity standardized by that in the control cells was assigned as 100%. Data were analyzed by one-way ANOVA and presented as mean ± SEM, ***P < 0.001 (right panel). **H** Huh7-NAT10 shRNA-1, Huh7-NAT10 shRNA-2, and Huh7-control shRNA cells treated with or without oxaliplatin. The cytoplasmic lysate and nuclear extraction were fractioned, which were subjected to WB using indicated antibodies. TOP I and α-tubulin were used as nuclear and cytoplasmic marker, respectively. **I** The nuclear acetyl-CoA levels in Huh7 cells expressing GFP, GFP-ACLY, GFP-ACLY K468R, and treated with oxaliplatin were detected. Data were analyzed by one-way ANOVA and presented as mean ± SEM (*n* = 3), ns denotes no significance, **P < 0.01. **J** The H3K27ac levels on the promoters of *PIK3R1* and *CYP2C9* genes were detected by ChIP in the Huh7 cells expressing GFP, GFP-ACLY, GFP-ACLY K468R and treated with oxaliplatin. Data were analyzed by one-way ANOVA and presented as mean ± SEM (*n* = 3), ns denotes no significance, ***P < 0.001.

To validate the existence of the endogenous ACLY K468-Ac in cells, we generated ACLY K468 acetylation specific antibody (designated as “ACLY K468-Ac”) to recognize the ACLY K468 acetylation. The dot blotting showed that ACLY K468-Ac antibody specifically recognizes acetyl-ACLY K468 peptide (Fig. 7A). Ectopic Flag-NAT10, but not Flag-NAT10 G641E enhanced the endogenous ACLY K468-Ac (Fig. 7B). The ACLY K468-Ac antibody detected the acetylated ACLY band in the immunoprecipitated GFP-ACLY but not GFP-ACLY K468R. In addition, ACLY K468-Ac level increased upon doxorubicin and oxaliplatin treatment (Fig. 7C and Fig. S9). Together, these data demonstrate that ACLY K468-Ac specifically recognizes endogenous K468-acetylated ACLY and the NAT10-mediated ACLY K468-Ac might function in response to DNA-damaging chemotherapy.

We further validated the cellular localization of ACLY K468-Ac upon DNA-damaging chemotherapy. Immunofluorescent staining showed that ACLY K468-Ac mainly localized in the nucleus and co-localized with NAT10 under doxorubicin or oxaliplatin treatment (Fig. 7D), indicating that the endogenous ACLY K468 is acetylated by NAT10 in the nucleus upon chemotherapy. This is further validated by the experiment showing that GFP-ACLY accumulated in the nucleus upon doxorubicin treatment (Fig. 7E). Surprisingly, we found that GFP-ACLY K468R and GFP-ACLY K468Q remain in the cytoplasm upon doxorubicin treatment (Fig. 7E), indicating that K468 is required for the nuclear localization of ACLY. The NLStradamus analysis showed that the residues 468–471 is predicted as the nuclear localization signal (NLS) of ACLY (Fig. 7F). These results demonstrate that K468 is a key residue which is responsible for the nuclear localization of ACLY, and is acetylated upon chemotherapy.

Further, ACLY K468-Ac significantly increased in the nucleus upon chemotherapy treatment only in the presence of NAT10 (Fig. 7G, H; Fig. S10), confirming that NAT10 acetylates ACLY K468 leading to the nuclear accumulation of ACLY upon DNA-damaging chemotherapy.

We thereafter explored the function of ACLY K468-Ac in the transcription of chemotherapy-resistant genes upon chemotherapy. Firstly, the nuclear acetyl-CoA level increased in Huh7 cells upon chemotherapy when GFP-ACLY but not GFP-ACLY K468R was expressed (Fig. 7I). Moreover, the levels of H3K27ac on *PIK3R1* and *CYP2C9* promoters were upregulated in the Huh7 cells expressing GFP-ACLY but not GFP-ACLY K468R (Fig. 7J). Taken together, these results indicate that ACLY K468-Ac is essential for the nuclear acetyl-CoA production and the activation of *PIK3R1* and *CYP2C9* gene transcription to drive chemoresistance.

### ACLY K468-Ac confers HCC cells and mouse xenografts chemoresistance

**Fig. 8:**
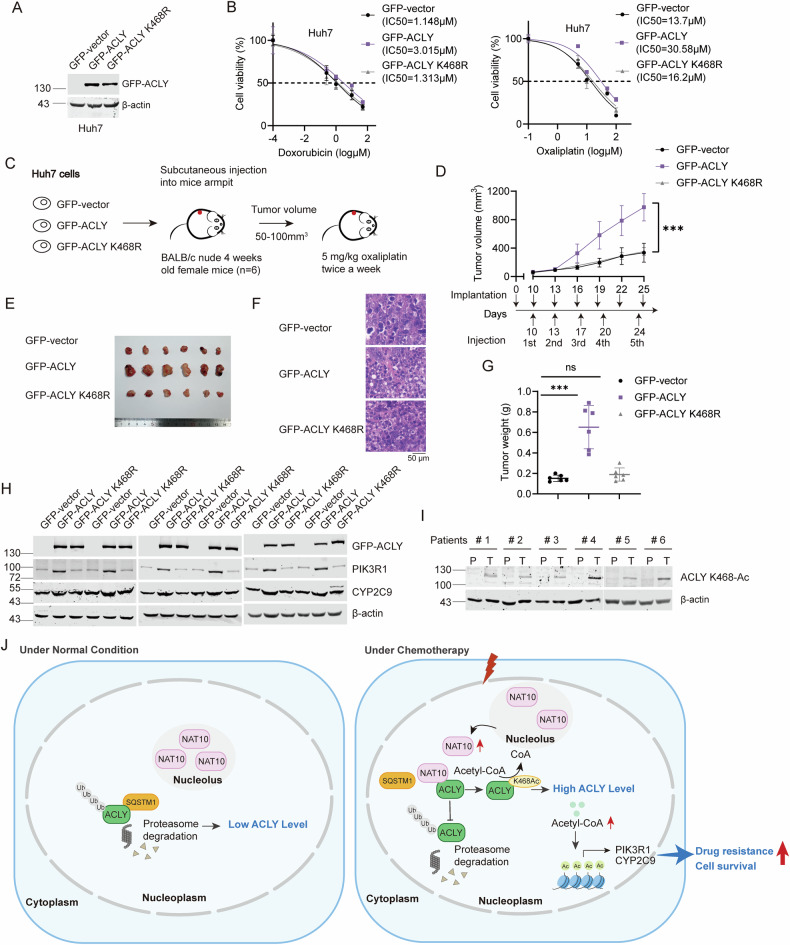
ACLY K468-Ac confers HCC cells and mouse xenografts chemoresistance. **A** WB was performed on the cell lysates to evaluate GFP-ACLY levels in GFP, GFP-ACLY, and GFP-ACLY K468R stable expression Huh7 cells. **B** The IC50 of doxorubicin and oxaliplatin in the GFP, GFP-ACLY, and GFP-ACLY K468R stable expression Huh7 cells. Data were analyzed by one-way ANOVA and presented as mean ± SD (*n* = 3). **C** GFP, GFP-ACLY, and GFP-ACLY K468R stable expression Huh7 cells were subcutaneously implanted into nude mice (*n* = 6). Oxaliplatin (5 mg/kg) was intraperitoneally injected twice a week after Huh7 cells injection. **D** The volumes of the tumor xenografts were shown, respectively. Data were analyzed by one-way ANOVA and presented as mean ± SD, ***P < 0.001. **E** Tumors were dissected at the end of the experiment. **F** HE staining was performed with these tumor tissues. Scale bar, 50 μm. **G** The weights of the tumor xenografts were shown, respectively. Data were analyzed by one-way ANOVA and presented as mean ± SD, ns denotes no significance, ***P < 0.001. **H** Proteins extracted from the xenografts were subjected to Western blot probed with anti-GFP-ACLY, anti-PIK3R1, and anti-CYP2C9 antibodies. Beta-actin was used as a loading control. **I** WB analysis of ACLY K468-Ac expression in 6 individual paired HCC tissues. **J** A working model explaining the mechanism by which NAT10 leads to chemoresistance.

To determine if ACLY K468-Ac controls chemoresistance in HCC cells, IC50 values of oxaliplatin and doxorubicin were determined in Huh7-GFP, Huh7-GFP-ACLY, and Huh7-GFP-ACLY K468R cells. The IC50 values of oxaliplatin and doxorubicin increased in the GFP-ACLY cells rather than that in the GFP-ACLY K468R cells (Fig. 8A, B), underlying that ACLY K468-Ac drives the chemoresistance in HCC. To further confirm the function of ACLY K468-Ac in the chemoresistance in vivo, we subcutaneously implanted Huh7-GFP, Huh7-GFP-ACLY, and Huh7-GFP-ACLY K468R cells into the BALB/c nude mice, and treated mice with oxaliplatin (Fig. 8C). The tumor volume and weight significantly increased in the Huh7-GFP-ACLY derived xenografts but not in those derived from Huh7-GFP-ACLY K468R (Fig. 8D–G), demonstrating that ACLY K468-Ac confers HCC cells and mouse xenografts resistance to chemotherapy. The expression of PIK3R1 and CYP2C9 in mouse xenografts is also upregulated via ACLY K468-Ac (Fig. 8H). In addition, ACLY K468-Ac level was upregulated in HCC tumor tissues compared with their non-tumorous counterparts (Fig. 8I). These results demonstrate that ACLY K468-Ac drives chemoresistance in HCC.

## Discussion

Chemotherapy has been used as the first-line treatment in the advanced HCC patients. Although chemotherapy-based HAIC effectively improves the survival of advanced HCC patients, only 46% patients can obtain clinical benefit due to the chemoresistance [4, 5]. However, the mechanisms involved in the chemoresistance in HCC are not fully understood. Here, we unravel that chemotherapy-induced acetylation of ACLY by NAT10 drives the chemoresistance in HCC through controlling nuclear acetyl-CoA production to activate the transcription of *CYP2C9* and *PIK3R1* genes (Fig. 8J).

NAT10, which is a key enzyme that acetylates both proteins and RNAs [24–27], was found to be upregulated in HCC and associated with poor prognosis of the patients [41]. However, the function of NAT10 in HCC chemoresistance remains unknown. Here we identify NAT10 as a potential driver of chemoresistance in HCC by functional validation in HCC cells and mouse xenografts in vivo. Furthermore, we found that NAT10 controls the mRNA levels of chemotherapy-resistant genes *CYP2C9* and *PIK3R1*. It is known that NAT10 regulates mRNA stability by acetylating mRNA at cytidine [27]. Previous study found that NAT10 drove the cisplatin resistance by promoting the mRNA stability of *AHNAK* in bladder cancer [47]. In the present study, we uncover a novel mechanism by which NAT10 drives chemoresistance in HCC. We show that the mRNA stabilities of *CYP2C9* and *PIK3R1* are not regulated by NAT10 upon chemotherapy, indicating that NAT10 promotes chemoresistance in HCC by activating the transcription of the downstream genes instead of modifying mRNA. This is validated by the ChIP experiments showing that the H3K27ac on the promoters of *CYP2C9* and *PIK3R1* is significantly upregulated by NAT10.

Next, we found that NAT10 activates the nuclear acetyl-CoA production upon chemotherapy in HCC cells. Acetyl-CoA is the sole donor of acetyl groups for histone and non-histone protein acetylation, and its crucial role in epigenetics has long been recognized. It is known that acetyl-CoA has energy-rich thioester bond which makes it energetically unstable, thus it is thought to be synthesized spatiotemporally within the subcellular compartment [17, 21, 48, 49]. Recent study found that the nonclassical nuclear TCA cycle exists in the nucleus to produce acetyl-CoA in situ [20]. The enzymes catalyzing acetyl-CoA production, including cytoplasmic pyruvate kinase M2 (PKM2) and mitochondrial pyruvate dehydrogenase complex (PDC), translocate into the nucleus to produce acetyl-CoA for facilitating histone acetylation in cancer and embryonic cells [17, 21, 48, 50]. Here we found that NAT10 specifically elevates the nuclear acetyl-CoA level without changing the cytoplasmic acetyl-CoA level upon chemotherapy, suggesting that NAT10 controls the production of nuclear acetyl-CoA in situ to activate gene transcription in response to DNA damage.

Furthermore, we elucidate the mechanism involved in the control of the nuclear acetyl-CoA production under chemotherapy. In the nucleus, acetyl-CoA production is controlled by three enzymes including PDC, ACLY, and ACSS2 [16, 18, 20]. Among these three acetyl-CoA production enzymes, ACLY level is upregulated to activate the production of nuclear acetyl-CoA in response to DNA damage, while nuclear PDC level increases in a cell-cycle-dependent manner and ACSS2 translocates from cytoplasm to nucleus under glucose deprivation. However, the mechanisms involved in the control of nuclear ACLY level upon DNA damage remains unknown [22]. Here, we firstly found that upon chemotherapy, ACLY was acetylated at K468 by NAT10. We further generated the ACLY K468-Ac specific antibody and show that the endogenous ACLY K468-Ac is specifically accumulated in the nucleus only in the presence of NAT10 upon chemotherapy. Additionally, GFP-ACLY K468R remains in the cytoplasm even under chemotherapy treatment, and K468–471 is predicted as the NLS of ACLY. Therefore, K468 is responsible for the nuclear entrance of ACLY, and its acetylation is essential for its nuclear accumulation upon chemotherapy.

It was reported that ACLY was acetylated at K540, K546, and K554 by p300/CBP-associated factor (PCAF), which blocked poly-ubiquitination at these sites and stabilized ACLY under high-glucose conditions, lipid synthesis, and tumor growth [51]. Here, we explored the mechanism of how ACLY K468-Ac controls its protein elevation. ACLY K468 acetylation inhibits the ubiquitin-proteasomal degradation of ACLY upon chemotherapy. It’s known that SQSTM1 is an E3 ubiquitin ligase for ACLY, which poly-ubiquitinates ACLY leading to the selective autophagic degradation of ACLY by lysosome pathways to maintain citrate homeostasis in ovarian granulosa cells [52]. Here, we found that SQSTM1 is among NAT10-binding proteins. Upon doxorubicin and oxaliplatin treatment, ACLY bound NAT10 in the nucleus, otherwise, it bound SQSTM1. Furthermore, loss of ACLY K468 acetylation enhanced the binding between ACLY and SQSTM1, indicating that ACLY K468-Ac abrogates SQSTM1-mediated ACLY ubiquitination in the nucleus, thus increases the nuclear ACLY level. Therefore, we unravel a novel mechanism controlling of nuclear ACLY level upon DNA damage.

Additionally, we further clarified the function of ACLY K468-Ac upon chemotherapy. GFP-ACLY K468R lost the capability of promoting the production of nuclear acetyl-CoA upon DNA damage chemotherapy, demonstrating that nuclear accumulation of ACLY K468-Ac is responsible for nuclear acetyl-CoA production in response to chemotherapy. GFP-ACLY K468R lost the capability of activating the transcription of *CYP2C9* and *PIK3R1* by H3K27ac, which sensitizes HCC cells and mouse xenografts to chemotherapy. Thus, blocking ACLY K468-Ac provides a potential therapeutic approach to reduce chemoresistance in HCC.

In the present study, we find that NAT10 is upregulated in response to DNA-damaging chemotherapy. The upregulation of NAT10 in HCC was found to be significantly correlated with the poor prognosis of HCC patients [41]. Our study implies that the high NAT10 level might impair the therapeutic response to chemotherapy-based HAIC in HCC patients. Clinically, NAT10 expression in biopsy specimens might help to guide individualized therapeutic strategies for advanced HCC patients. In addition, our study elucidates the important role of NAT10-mediated ACLY K468-Ac in chemoresistance. Thus, small-molecule inhibitors that block the ACLY K468-Ac might be a promising therapeutic strategy for reducing NAT10-related chemoresistance in HCC. Recent studies also showed that the combination of lenvatinib, PD-1 antibody and chemotherapy-based HAIC significantly improved the clinical outcome in HCC patients compared with monotherapy [53]. In this combinational therapy, effective chemotherapy enhances the therapeutic efficacy of the PD-1 antibody [54–56]. Since ACLY K468-Ac drives chemoresistance in HCC, therefore blocking of ACLY K468-Ac will provide an opportunity to enhance the efficacy of chemo-immunotherapy in HCC.

It has recently found that chemotherapeutic drugs including paclitaxel, doxorubicin, oxaliplatin or cisplatin, exacerbated metastasis in breast cancer, pancreatic cancer, colorectal cancer or gastric cancer [57–61], while the involved mechanisms remain elusive. It was previously found that NAT10 promotes metastasis in HCC through epithelial-mesenchymal transition (EMT) [62], and contributes to doxorubicin chemoresistance in HCC by regulating chemotherapy-induced EMT with unknown mechanism [43]. However, the mechanism by which NAT10 regulates chemotherapy-induced metastasis in HCC remains unknown. It was reported that acetyl-CoA activates the transcription of *TWIST2* to regulate EMT in HCC [63], we thus anticipate that NAT10 might facilitate the chemotherapy-induced metastasis in HCC through increasing acetyl-CoA production. Therefore, targeting NAT10 might alleviate the occurrence of chemotherapy-induced metastasis in addition to sensitize the response of HCC to chemotherapy. However, this hypothesis needs further validation.

In conclusion, our study uncovers that chemotherapy-induced acetylation of ACLY K468 by NAT10 drives HCC chemoresistance. The inhibition of the NAT10-ACLY K468-Ac axis provides a promising strategy for strengthening the efficacy of chemotherapy in advanced HCC patients.

## Material and methods

### Cell culture and transfection

Huh7, HeLa, and HEK 293T cells were maintained in DMEM supplemented with 10% fetal bovine serum. All cell lines were purchased from the cell bank of the Chinese Academy of Medical Sciences and were routinely tested for Mycoplasma contamination.

Cells were transfected with plasmid DNA, shRNA, and siRNA duplexes using Lipofectamine 2000 (Invitrogen) according to the manufacturer’s protocol. In transient transfection experiments, plasmid DNA concentrations were maintained at a constant level with an empty vector. Small interfering RNAs (siRNAs) targeting NAT10 or control siRNA were synthesized (GenePharma). The sequences of all shRNAs and siRNAs were listed in Supplementary Table 1.

### Plasmids, stable cell lines, and oxaliplatin-resistant Huh7 cells construction

Flag-NAT10, Flag-NAT10 G641E, GST-NAT10, and its mutant plasmids were generated in our laboratory. GFP-ACLY and GFP-ACLY mutants were cloned into the pLV-EGFP vector. GST-ACLY and GST-ACLY mutants were cloned into the pGEX-4T1 vector. His-ACLY were cloned into the pET-28b (+) vector. All plasmids cloned with PCR inserts were confirmed by DNA sequencing.

The NAT10 knockout and control HeLa cell lines constructed by CRISPR-Cas9 genome editing technology were generated in our laboratory. The NAT10 stable knockdown and control HeLa or Huh7 cell lines were constructed by short hairpin RNA (shRNA)-mediated knockdown. Briefly, NAT10 shRNA-1, NAT10 shRNA-2, and control shRNA oligos were cloned into the pLKO.1 plasmid to obtain a lentiviral particle, and the HeLa or Huh7 cell lines were infected by the indicated lentivirus. The NAT10 stable knockdown cells were screened by puromycin. NAT10 knockdown was confirmed by Western blot. The pLV-EGFP-vector, pLV-EGFP-ACLY, and pLV-EGFP-ACLY K468R plasmids were used in the lentiviral particle packaging. Huh7 cell lines were infected with the lentiviral particle to obtain cells stably expressing GFP, GFP-ACLY or GFP-ACLY K468R.

For oxaliplatin-resistant Huh7 cells construction, Huh7 cells were treated with 50 μM oxaliplatin for 8 h, and then oxaliplatin was removed. Surviving cells were maintained in DMEM supplemented with 10% fetal bovine serum.

### Antibodies and reagents

Anti-ACLY (A3719, 1:3000), anti-PIK3R1(A4992, 1:1000), anti-AKT (A22412, 1:3000), anti-H3K27ac (A7253, 1:500), anti-β-actin (AC004, 1:5000), anti-α-tubulin (AC012, 1:5000), anti-TOP 1 (A12409, 1:2000) were purchased from ABclonal. Anti-CYP2C9 (bs-2887R, 1:1000) was purchased from Bioss. Anti-Flag (HT201, 1:1000) and anti-GFP (HT801, 1:2000) were purchased from Transgen. Anti-p-AKT (ab81213, 1:1000) was purchased from Abcam. Anti-acetylated lysine (9441, 1:1000) was purchased from Cell Signaling Technology. Anti-NAT10 was a gift from Dr. Bo Zhang. For generating anti-ACLY K468-Ac specific antibody, the peptide “SRADEVAPAK (ac) KAKPAMPQ” was synthesized and used to immunize the New Zealand rabbit by Beijing Jiaxuan Zhirui Biotechnology.

Oxaliplatin (OXA, 61825-94-3) was purchased from Aladdin. Doxorubicin (DOX, HY-15142A), Cycloheximide (CHX, HY-12320), and MG132 (HY-13259) were purchased from MedChemExpress.

### Cell fractionation experiment

Cellular fractionation was performed as described previously [35]. Briefly, cells were collected and resuspended in Buffer A (1 mM HEPES-KOH pH 7.9, 1.5 mM MgCl_2_, 10 mM KCl, 0.5 mM DTT, 0.5% NP-40, and proteinase inhibitors), and centrifugated to collect supernatant as cytoplasmic fraction. The pellet was washed with Buffer A and suspended in Buffer T (1% NP-40, 450 mM NaCl, 50 mM Tris-Cl pH 7.4, 1 mM PMSF, 0.2 mM Na_3_VO_4_, 5 mM β-glycerol phosphate, 20% glycerol, 2 mM DTT and proteinase inhibitors). The supernatant was collected as a nuclear fraction after centrifugation.

### Co-immunoprecipitation experiment

Cells were collected and resuspended in Buffer A (25 mM Tris-Cl pH 7.5, 150 mM KCl, 1 mM DTT, 2 mM EDTA, 0.5 mM PMSF and 0.5% NP-40 and proteinase inhibitors) and then were sonicated. The supernatant was collected after centrifugation and used for immunoprecipitation. Antibodies were coupled with protein A-Sepharose beads (GE Healthcare) in IPP500 (500 mM NaCl, 10 mM Tris-Cl pH 8.0, 0.5% NP-40), and the coupled beads were incubated with cell supernatant. After washing, the precipitants were analyzed by Western blot using the indicated antibodies.

### Real-time PCR

Real-time PCR was performed as described previously [28]. Total RNA was extracted from cells using TRIzol reagent (Invitrogen). The cDNA was synthesized by these RNA reverse transcription and real-time PCR was performed. The human β-actin was used as control. All real-time PCR data were analyzed by comparative Ct method and normalized to β-actin. The sequences of all primes were listed in Supplementary Table 1.

### Chromatin immunoprecipitation

1% formaldehyde was added to cells for cross-linking nuclear proteins with genomic DNA, and 0.125 M glycine was added to stop the cross-linking. The cells were collected by centrifugation and resuspended in ChIP buffer (50 mM HEPES-KOH pH 7.5, 140 mM NaCl, 1% Triton X-100, 0.1% Sodium Deoxycholate, 0.1% SDS, 1 mM EDTA, and protease inhibitors). Genomic DNA was sonicated to get a length of approximately 300–1000 bp. Cell supernatants were collected by centrifugation, diluted by 1:10 in RIPA buffer (50 mM Tris-HCl pH 8.0, 150 mM NaCl, 2 mM EDTA pH 8.0, 1% NP-40, 0.5% Sodium Deoxycholate, 0.1% SDS and protease inhibitors) and incubated with anti-H3K27ac or control IgG for 1 h at 4 °C with rotation. Protein A-Sepharose beads were added to each reaction and incubated overnight at 4 °C with rotation. Protein A-Sepharose beads were collected by centrifugation and performed the following washes: once in low salt wash buffer (0.1% SDS, 1% Triton X-100, 2 mM EDTA, 150 mM NaCl and 20 mM Tris-HCl pH 8.0), once in high salt wash buffer (0.1% SDS, 1% Triton X-100, 2 mM EDTA, 500 mM NaCl and 20 mM Tris-HCl pH 8.0) and once in LiCl wash buffer (0.25 M LiCl, 1% NP-40, 1% sodium deoxycholate, 1 mM EDTA and 10 mM Tris-HCl pH 8.0). Elute DNA by adding elution buffer to the protein A beads and vortex slowly for 15 min at 30 °C. The protein-DNA cross-linking was reversed by incubation with 200 mM NaCl, RNase A, and proteinase K at 65 °C. The DNA was purified using phenol: chloroform extraction and subjected to real-time PCR. The sequences of all primes were listed in Supplementary Table 1.

### Protein purification

The experiment was performed as described previously [28]. Flag-NAT10-His were cloned into pFast-Bac1. The recombinant baculoviruses were generated with the Bac-to-Bac Baculovirus Expression System (Invitrogen). Flag-NAT10-His proteins were purified from baculovirus-infected *sf*9 cells using Ni-NTA agarose (Qiagen) according to the manufacturer’s instructions. His-ACLY and GST recombinant proteins were expressed in *E. coli* strain BL21 (DE3), which treated with isopropyl-β-D-thiogalactoside to induce fusion protein expression. Cells were lysed by sonication and then purified using the Glutathione Sepharose 4B (GE Healthcare) for GST proteins or using Ni-NTA agarose (Qiagen) for His-ACLY proteins, respectively. Purified proteins were separated on SDS-PAGE followed by Coomassie blue staining or Western blot.

### GST pull-down experiment

GST fusion proteins were prepared following standard protocol. For in vitro binding experiments, GST fusion proteins bound to the Glutathione Sepharose 4B (GE Healthcare) were incubated with purified proteins. After washing, the bound proteins were separated by SDS-PAGE and immunoblotted with indicated antibodies.

### In vitro acetylation experiment

Purified GST-ACLY and Flag-NAT10 proteins were incubated in the reaction buffer (50 mM Tris-Cl pH 7.9, 10% glycerol, 0.1 mM EDTA, 1 mM PMSF, 10 mM sodium butyrate, 10 μM acetyl-CoA) at 30 °C for 2 h. Reaction mixtures were separated by SDS-PAGE and subjected to Western blot probed with anti-acetyl-lysine antibody.

### Immunofluorescent staining

Cells were seeded on slide in 24-well plate. After fixed with 4% paraformaldehyde for 15 min, cells were permeabilized using 0.5% Triton X-100 for 20 min at room temperature. Cells were incubated with 10% goat serum for 30 min at 37 °C. Primary antibodies were incubated with the cells at 4 °C overnight. After cells were washed three times with PBS, TRITC-conjugated anti-rabbit antibody, FITC-conjugated anti-mouse antibody and DAPI were incubated at 37 °C for 30 min. Finally, cells were washed with PBS and sealed. Photos were taken under confocal microscopy (TCS SP8 DIVE MP FLIM, Leica, Germany).

### Identification of acetylation site in ACLY by LC–MS/MS analysis

In vitro, acetylation experiment was performed using purified GST-ACLY and Flag-NAT10 proteins. The reaction was resolved by SDS-PAGE and visualized by Coomassie blue staining. The bands were cut from SDS-PAGE, fully trypsinized, and analyzed by Q-Extractive liquid chromatography-tandem mass spectrometry (LC–MS/MS) using mass spectrometer Orbitrap Velos Pro (Thermo). Mass spectrometry was carried out and data were processed using the Proteome Discoverer software (Version 1.4).

### Acetyl-CoA concentration measurement

Acetyl-CoA concentration was measured using the acetyl-CoA assay kit (Bioss, AK341) according to the instruction. In brief, cells were collected, sonicated, and centrifuged. The supernatant was mixed with the buffer containing malate dehydrogenase and citrate synthase in a 96-well plate. Since the acetyl-CoA content is proportional to the rate of production of nicotinamide adenine dinucleotide (NADH) in the coupling reaction of malate dehydrogenase and citrate synthase, the acetyl-CoA content is determined by measuring the absorbance of NADH at 340 nm. The absorbance at 340 nm measured at 20 s was used as value A1, and that measured at 80 s was used as value A2. and ΔA = A2-A1 was calculated, which was converted by the formula [acetyl-CoA content (nmol/10^4^) = (3280 × ΔA + 0.024) ÷ 500] to obtain the level of acetyl-CoA.

### IC50 determination

Cell growth was analyzed using CCK8 reagent (Yeasen) according to the manufacturer’s direction. Huh7 cells were plated on 96-well plates and treated with indicated concentration of oxaliplatin and doxorubicin for 48 h or 72 h. Cells were washed with PBS. CCK8 diluted by DMEM was added to cells and incubated for 2 h at 37 °C, and absorbance at 450 nm was evaluated.

### Nude mice xenograft models

4-week-old BALB/c nude female mice were randomly separated into 3 groups (*n* = 6, per group). Huh7-control-shRNA, Huh7-NAT10-shRNA-1, Huh7-NAT10-shRNA-2 cells or Huh7-GFP, Huh7-GFP-ACLY, Huh7-GFP-ACLY K468R cells (5 × 10^6^) were injected subcutaneously into the right flanks in each mouse, respectively. The tumor volume was measured every three days and calculated using the formula length × width^2^ × 0.5 (mm^3^). When the tumor volume reaches 50–100 mm^3^, 5 mg/kg oxaliplatin was intraperitoneally injected twice a week. The mice were sacrificed when the maximum diameter of the tumor reaches 1.5 cm, and the tumor volume and weight were measured. Animal operations were performed according to the National Institutes of Health guidelines. Studies on animals obtained the approval of the Institutional Animal Care and Use Committee of Peking University Health Science Center (Ethics Approval License: BCJE0165).

### Human tissue samples

HCC and the adjacent non-tumorous tissues used for Western Blot were obtained from the patients who underwent tumor resection at Peking University Cancer Hospital (PUCH). Written informed consent was obtained from the participating patients. The informed consent and experimental procedures were approved by the ethics committee of the Peking University Health Science Center (2006021).

### Database

The IHC data and the related clinical data of HCC were obtained from HPA website (https://www.proteinatlas.org/).

### Statistical analysis

Statistical analysis was performed using SPSS software version 26.0 (IBM, Armonk, NY, USA). The post hoc analysis, LSD (Least Significant Difference) test for one-way ANOVA was used to analyze the differences among more than two groups. The two groups were analyzed using *T*-tests. The group size is the minimum number that is sufficient to define a functional parameter and the variance is similar between the groups. All data met the assumptions of the tests and represented in the figures with the error of the mean (mean ± SEM or SD). *P* < 0.05 was considered statistically significant.

### Supplementary information

Supplementary Figures and Table

Original Western blots

## Data Availability

The datasets used and/or analyzed during the present study are available from the corresponding author on reasonable request.
