# Supplementary material for: Chemotherapy-induced acetylation of ACLY by NAT10 promotes its nuclear accumulation and acetyl-CoA production to drive chemoresistance in hepatocellular carcinoma
Source: Cell Death Dis. 2024 Jul 31;15(7):545. doi: 10.1038/s41419-024-06951-9 (PMC11291975; doi:10.1038/s41419-024-06951-9)
Supplement: Supplementary file 1 — Supplementary Figures and Table [file 41419_2024_6951_MOESM1_ESM.docx]

**Supplementary Figures**


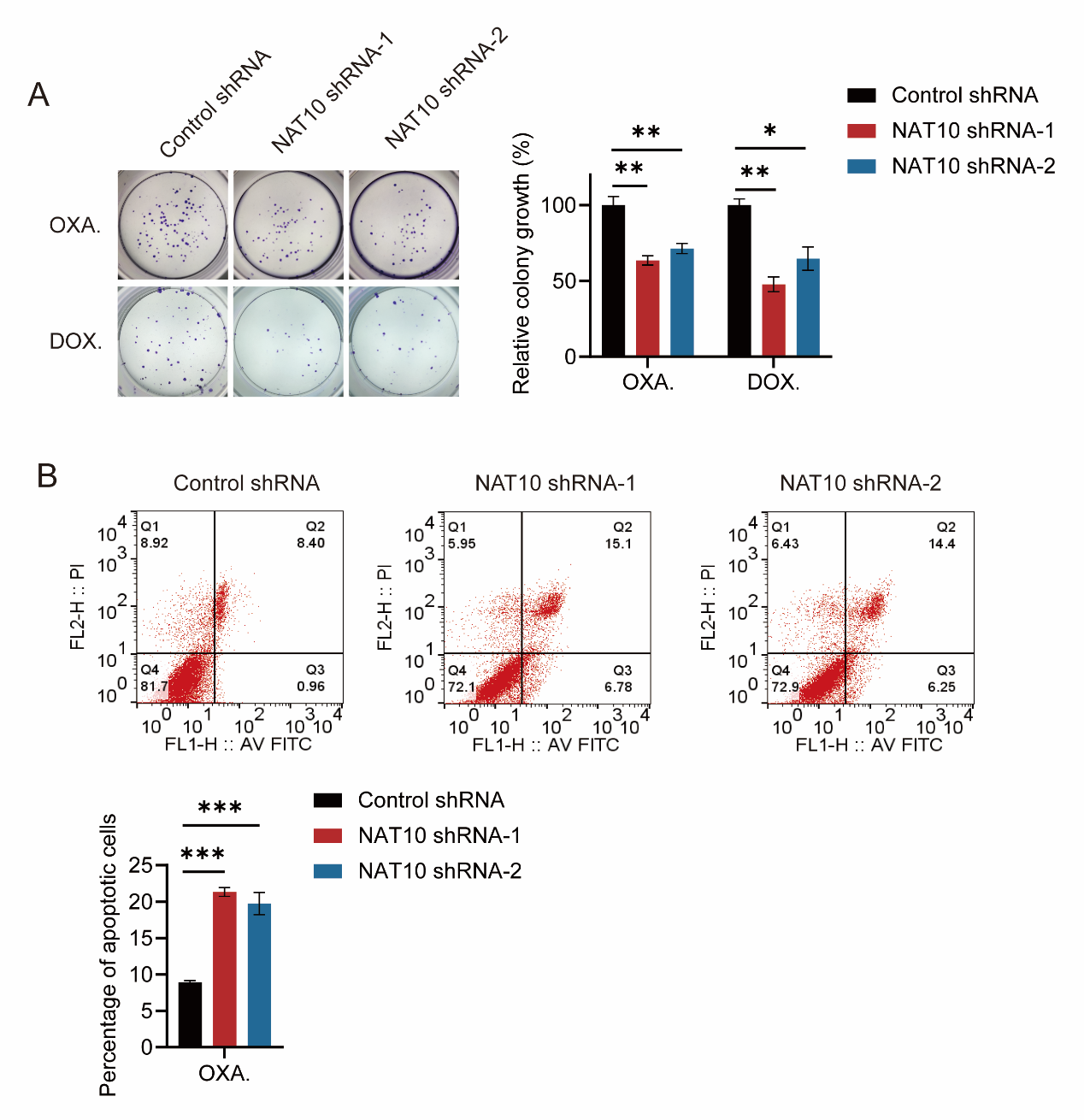


**Supplementary Figure 1. Targeting NAT10 promotes HCC cells growth inhibition and apoptosis under chemotherapy. A** Huh7-NAT10 shRNA-1, Huh7-NAT10 shRNA-2 and Huh7-control shRNA cells were treated with IC50 concentrations chemotherapeutic drugs (oxaliplatin OXA, 12 μM and doxorubicin DOX,1.2 μM). Colony formation assay was performed. Data were analyzed by one-way ANOVA and presented as mean±SEM, **P* < 0.05, ***P* < 0.01. **B** Huh7-NAT10 shRNA-1, Huh7-NAT10 shRNA-2 and Huh7-control shRNA cells were treated with 12 μM OXA. Apoptotic cells were determined by flow cytometry. Data were analyzed by one-way ANOVA and presented as mean±SEM, ****P* < 0.001.


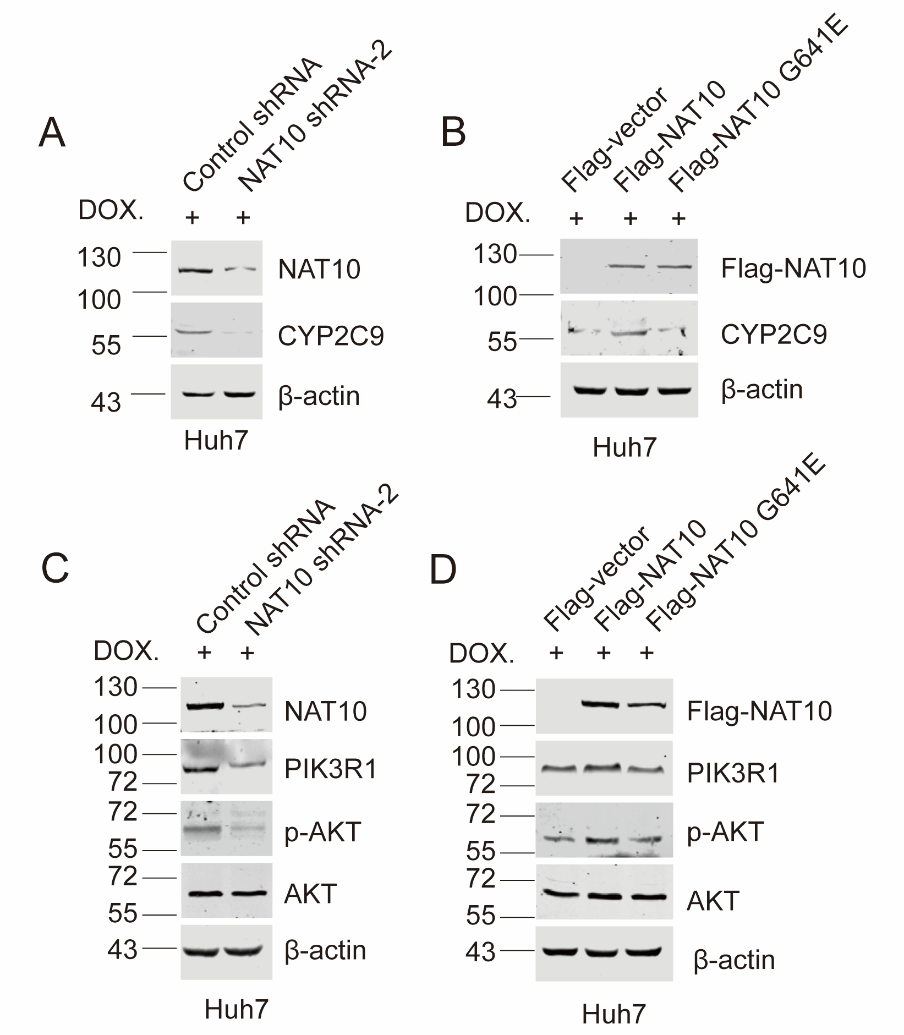


**Supplementary Figure 2. NAT10 regulates the protein levels of PIK3R1, p-AKT, AKT and CYP2C9 depending on its acetyltransferase activity. A**, **C** The protein levels of PIK3R1, p-AKT, AKT and CYP2C9 were detected by WB in Huh7-NAT10 shRNA-2 and Huh7-control shRNA cells with doxorubicin treatment. **B**, **D** Huh7 cells were transfected with Flag-vector, Flag-NAT10 or Flag-NAT10 G641E plasmids and treated with doxorubicin. The protein levels of PIK3R1, p-AKT, AKT and CYP2C9 were detected by WB.


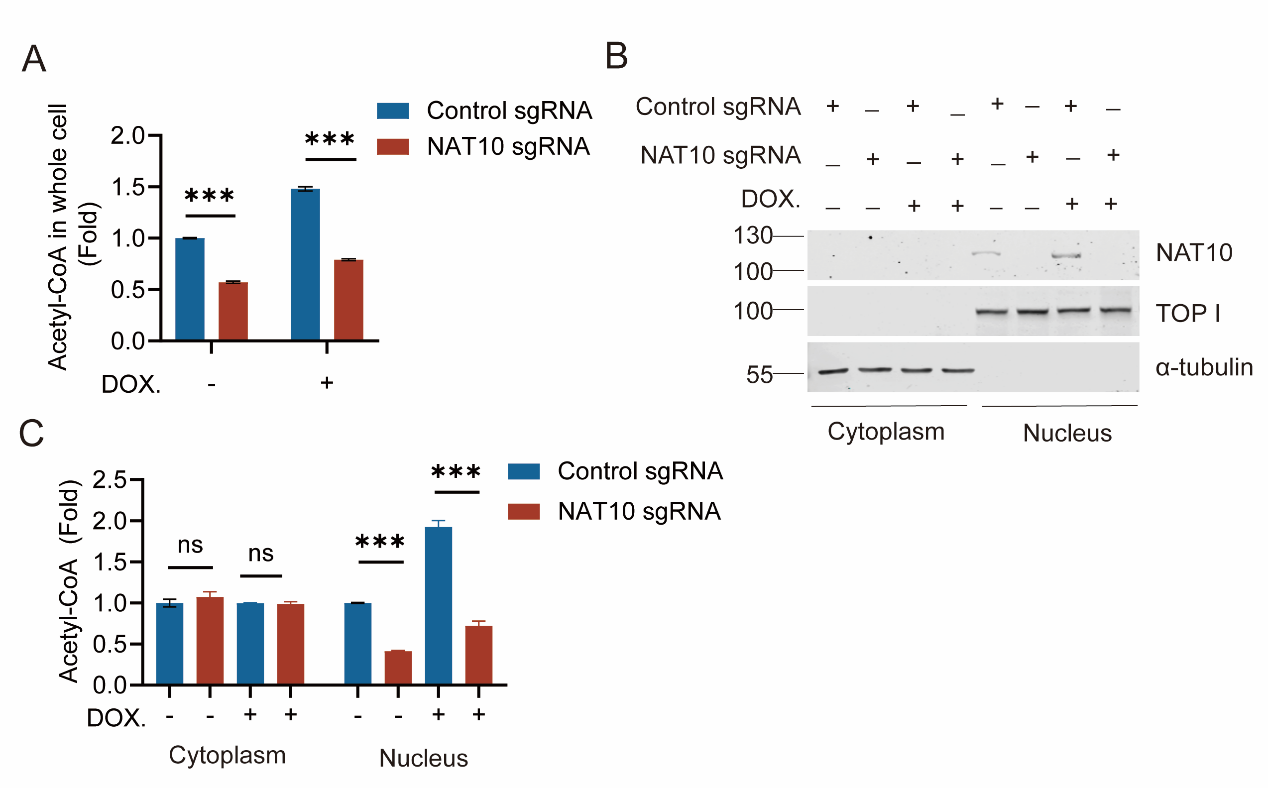


**Supplementary Figure 3. NAT10 controls the acetyl-CoA levels in HeLa cells.** **A** The acetyl-CoA levels were analyzed in NAT10 sgRNA and control sgRNA cells with or without doxorubicin treatment. Data were analyzed by one-way ANOVA and presented as mean±SEM, ****P* < 0.001. **B** Cellular fraction was prepared in NAT10 sgRNA and control sgRNA cells with or without doxorubicin treatment. **C** The acetyl-CoA levels were analyzed in **B**’s cellular fractions. Data were analyzed by one-way ANOVA and presented as mean± SEM, ns denotes no significance, ****P* < 0.001.


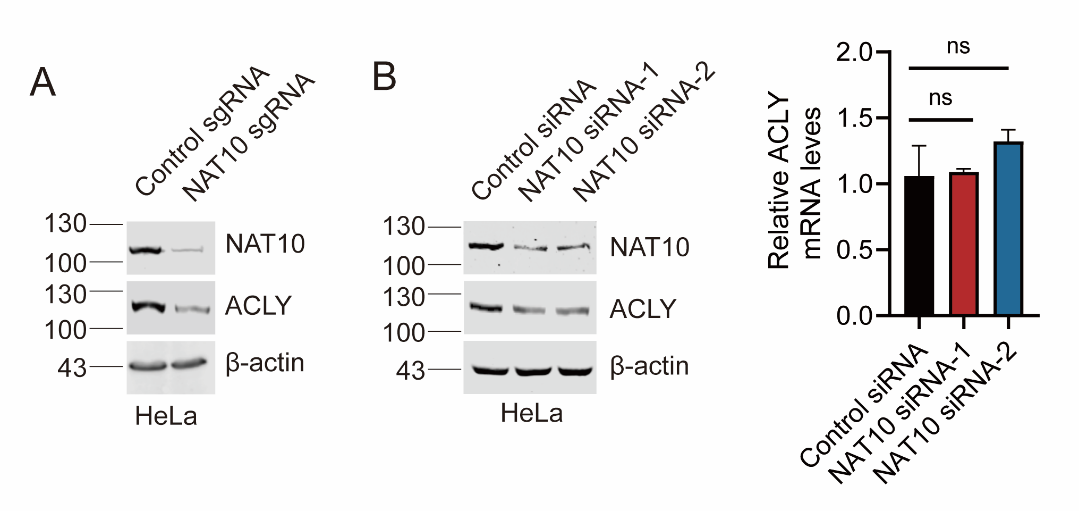


**Supplementary Figure 4. NAT10 controls ACLY protein levels and does not affect ACLY mRNA levels in HeLa cells. A** WB was performed on the cell lysates to evaluate NAT10 and ACLY protein levels in HeLa-NAT10 sgRNA and HeLa-control sgRNA cells. **B** HeLa cells were transfected with NAT10 or control siRNAs. WB was performed on the cell lysates to evaluate NAT10 and ACLY protein levels. ACLY mRNA level was evaluated by RT-qPCR. Data were analyzed by one-way ANOVA and presented as mean±SEM (*n*=3), ns denotes no significance.


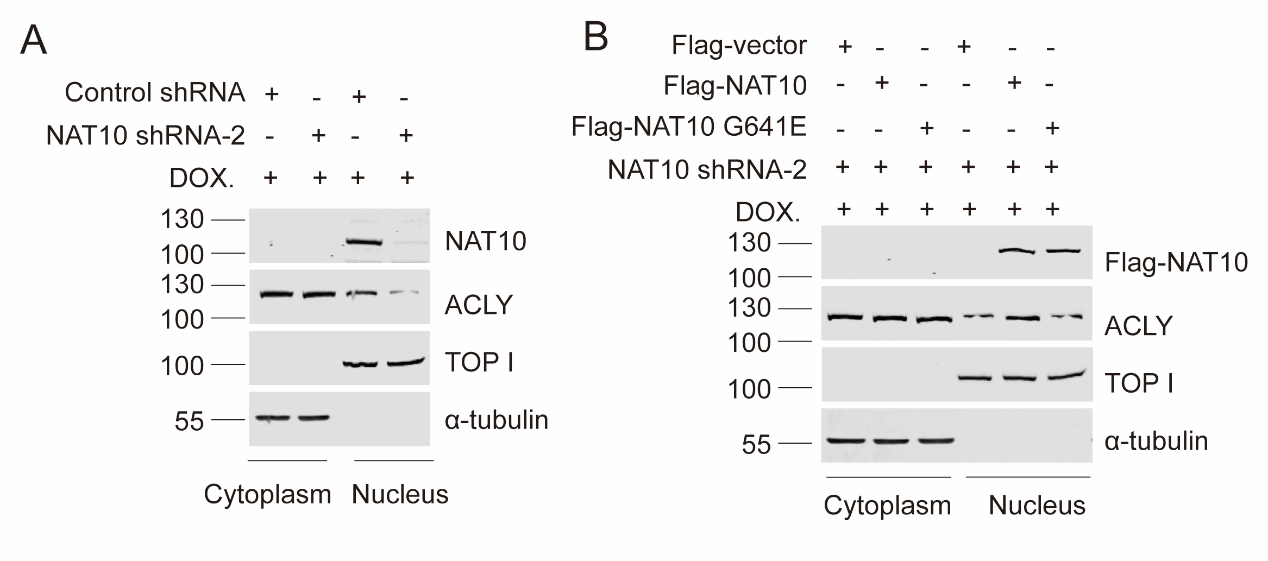


**Supplementary Figure 5. NAT10 controls nuclear ACLY protein levels with doxorubicin treatment. A** NAT10 shRNA-2 and control shRNA cells treated with 5 μM doxorubicin. The cytoplasmic lysate and nuclear extraction were fractioned, which were subjected to WB using indicated antibodies. TOP I and α-tubulin were used as nuclear and cytoplasmic marker, respectively. **B** NAT10 shRNA-2 cells were transfected with Flag-vector, Flag-NAT10 G641E, Flag-NAT10 plasmids and treated with 5 μM doxorubicin. The cytoplasmic lysate and nuclear extraction were fractioned, which were subjected to WB using indicated antibodies. TOP I and α-tubulin were used as nuclear and cytoplasmic marker, respectively.


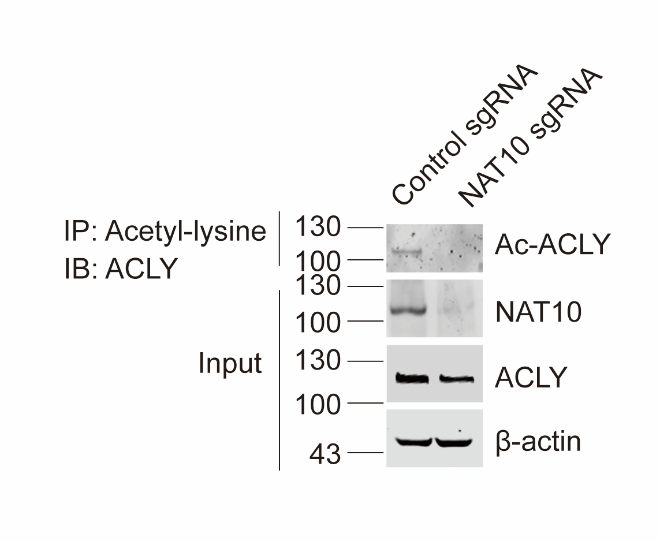


**Supplementary Figure 6. The acetylation levels of ACLY decreased after NAT10 was deleted.** HeLa-NAT10 sgRNA and HeLa-control sgRNA cells were subjected to immunoprecipitation using anti-acetyl-lysine. The acetylation levels of ACLY were evaluated by WB using anti-ACLY.


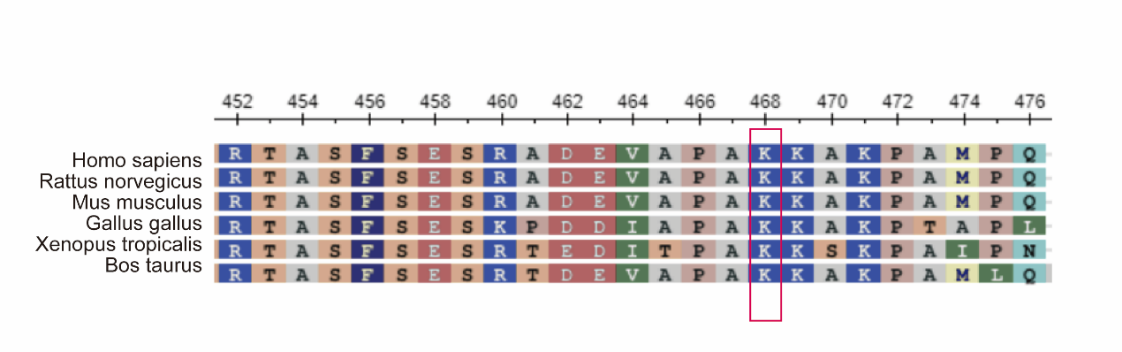


**Supplementary Figure 7. ACLY K468 site was found to be conserved among multiple species.** Alignment of ACLY amino acid sequences in different species showed the conservation of K468.


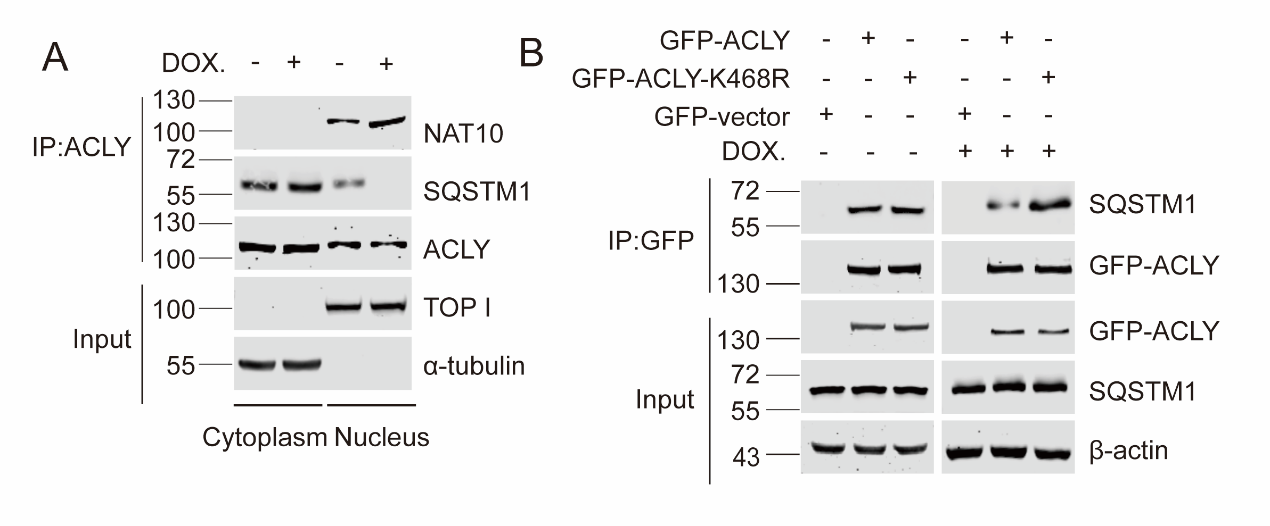


**Supplementary Figure 8. NAT10 competes with SQSTM1 to bind ACLY in the nucleus under DNA damage chemotherapeutic drug treatment.** **A** Huh7 cells were treated with or without doxorubicin. The cytoplasmic lysate and nuclear extraction were fractioned, which were subjected to immunoprecipitation using anti-ACLY antibody. The immunoprecipitates were subsequently immunoblotted with the indicated antibodies. TOP I and α-tubulin were used as nuclear and cytoplasmic marker, respectively. **B** Huh7 cells were transfected with indicated plasmids. Proteins were immunoprecipitated using anti-GFP. The immunoprecipitates were subsequently immunoblotted with the indicated antibodies.


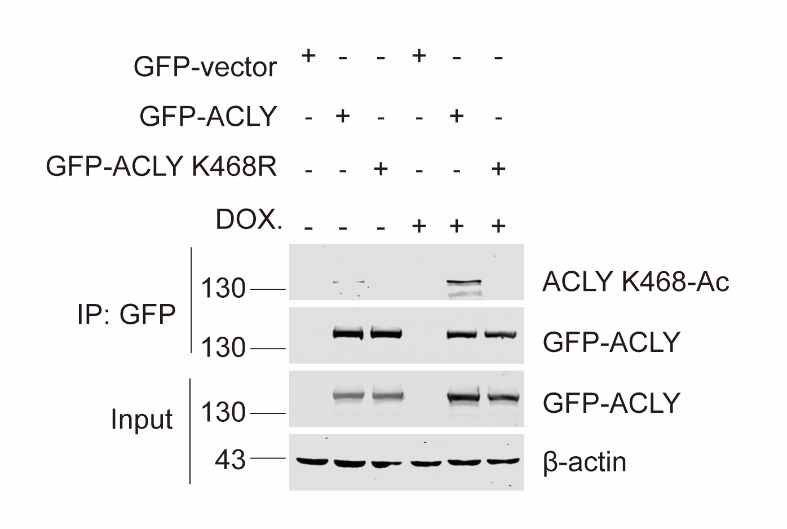


**Supplementary Figure 9. ACLY K468-Ac levels were upregulated under doxorubicin treatment.** Huh7 cells were transfected with GFP-vector, GFP-ACLY, GFP-ACLY K468R plasmids and treated with doxorubicin. Co-immunoprecipitation was performed with anti-GFP and the acetylation levels of GFP-ACLY were evaluated by WB using anti-ACLY K468-Ac.


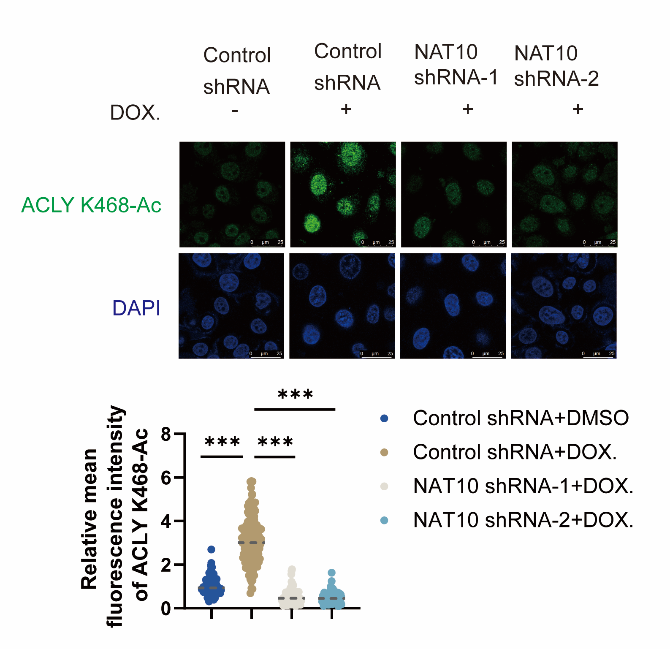


**Supplementary Figure 10. NAT10 controls ACLY K468-Ac level under DOX-induced DNA damage.** Immunofluorescent staining showed the fluorescent intensity of ACLY K468-Ac (green) in NAT10 knockdown or control cells treated with DMSO or doxorubicin. Scale bar, 25 μm (upper panel). The fluorescent intensity of ACLY K468-Ac was determined by scanning with Leica LAS X confocal software (*n*= 100~120). The relative fluorescent intensity standardized in the control cells was assigned as 100%. Data were analyzed by one-way ANOVA. ****P <*0.001 (lower panel).

**Supplementary Table 1**

| **The primers used in present study** | | |
| --- | --- | --- |
| **Primer names** | **Forward (5'-3')** | **Reverse (5'-3')** |
| CYP2C9(RT-qPCR) | CAGAGACGACAAGCACAACCCT | ATGTGGCTCCTGTCTTGCATGC |
| CYP2C19(RT-qPCR) | CATGGATATGAAGTGGTGAAG | TCCATTGCTGAAAACGATTC |
| PIK3R1(RT-qPCR) | TGGACGGCGAAGTAAAGCATT | AGTGTGACATTGAGGGAGTCG |
| XRCC1(RT-qPCR) | ACGAATGCCAGGGAGGGGTTGTC | AGCGGTGGCAGCGGAGATGAAG |
| NEIL1(RT-qPCR) | GCCCTATGTTTCGTGGACATC | CGCTAGGTTTCGTAGCACATTC |
| β-actin(RT-qPCR) | CATGTACGTTGCTATCCAGGC | CTCCTTAATGTCACGCACGAT |
| PIK3R1(CHIP) | TGCGAGTTGCAATCGACCT | AGGGCATCCTCGGACGAATA |
| CYP2C9(CHIP) | ACAGAGTGGACAATGGAACGAAGG | TGCTCCTTCTGAAAGGACTTTGACCC |
|  |  |  |
| **Sequences of RNA Oligonucleotides** | | |
| **Name** | **Sense strand (5’-3’)** | **Antisense strand (5’-3’)** |
| **siRNA** |  |  |
| NAT10siRNA-1 (CDS) | GGCCAAAGCUGUCUUGAAA | UUUCAAGACAGCUUUGGCC |
| NAT10siRNA-2(3'UTR) | UUGCCACGAGUCUCUCUCUUC | GAAGAGAGAGACUCGUGGCAA |
| SQSTM1siRNA | CGCUCACCGUGAAGGCCUATT | UAGGCCUUCACGGUGAGCGTT |
| **shRNA** |  |  |
| NAT10shRNA-1(CDS) | GAGAUGUAUUCACGGAAUAUG | CAUAUUCCGUGAAUACAUCUC |
| NAT10shRNA-2 (3'UTR) | UUGCCACGAGUCUCUCUCUUC | GAAGAGAGAGACUCGUGGCAA |
