# Supplementary material for: Chemotherapy-induced acetylation of ACLY by NAT10 promotes its nuclear accumulation and acetyl-CoA production to drive chemoresistance in hepatocellular carcinoma
Source: Cell Death Dis. 2024 Jul 31;15(7):545. doi: 10.1038/s41419-024-06951-9 (PMC11291975; doi:10.1038/s41419-024-06951-9)
Supplement: Supplementary file 2 — Original Western blots [file 41419_2024_6951_MOESM2_ESM.pdf]

Figure 1 C

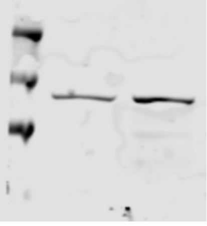
NAT10

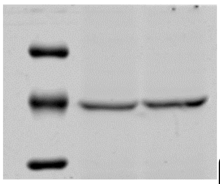
β-actin

Figure 1 D

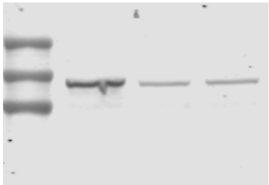
NAT10

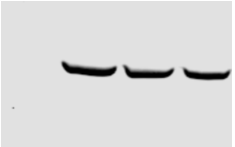
β-actin

Figure 1 F

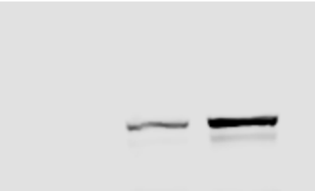
Flag-NAT10

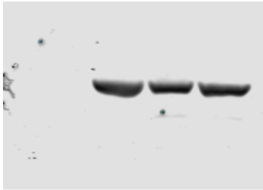
β-actin

Figure 2E

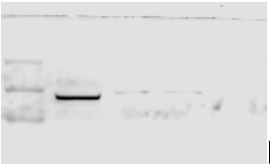
NAT10

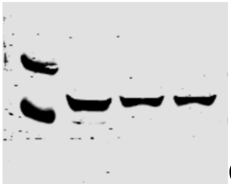
CYP2C9

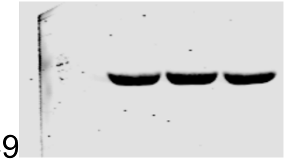
β-actin

Figure 2 F

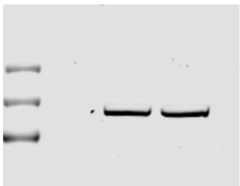
Flag-NAT10

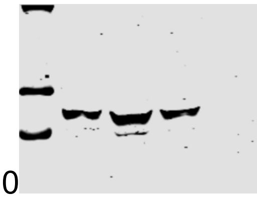
CYP2C9

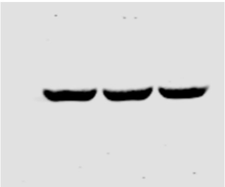
β-actin

Figure2G

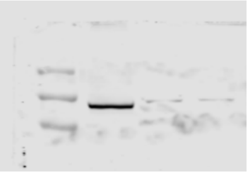
NAT10

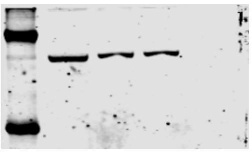
PIK3R1

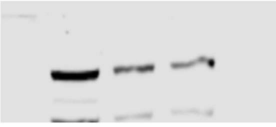
p-AKT

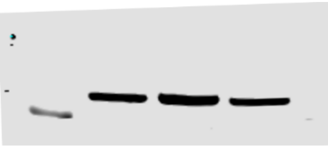
AKT

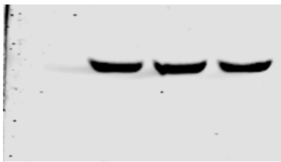
β-actin

Figure2H

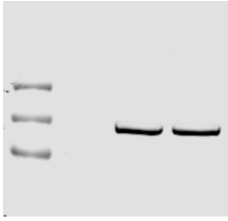
Flag-NAT10

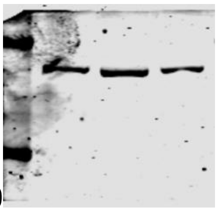
PIK3R1

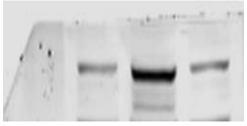
p-AKT

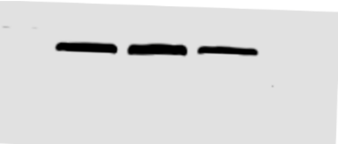
AKT

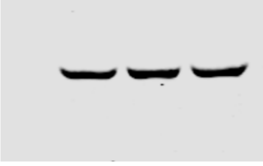
β-actin

Figure2K

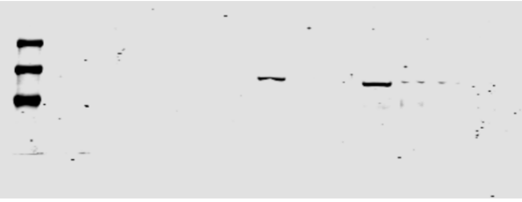
NAT10

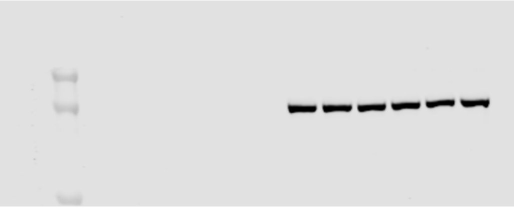
TOP1

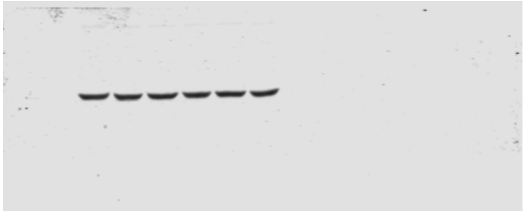
α-tubulin

Figure 3C

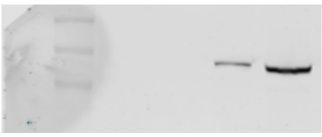
ACLY-IP

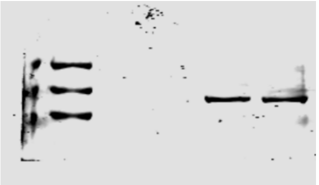
NAT10-IP

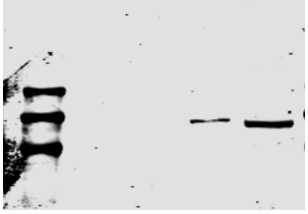
NAT10-input

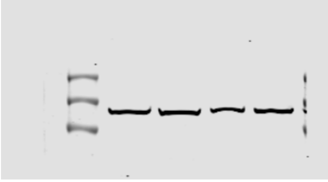
ACLY-input

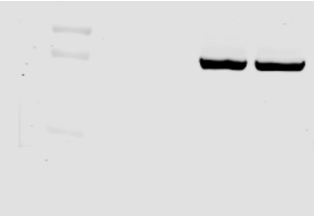
Top1-input
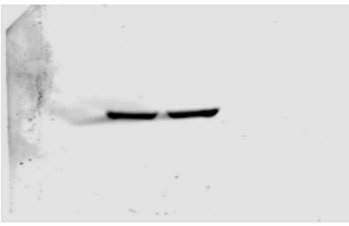
α-tubulin

Figure 3D

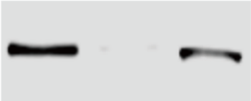
Flag-NAT10

Figure 3E

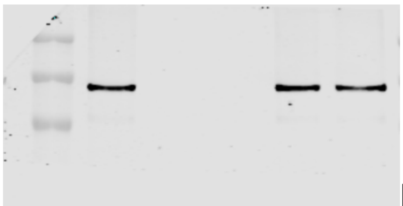
His-ACLY

Figure 3F

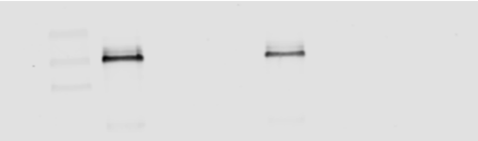
Flag-NAT10

Figure 4A

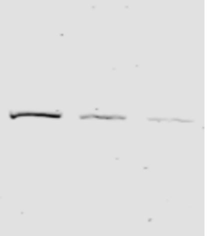
NAT10
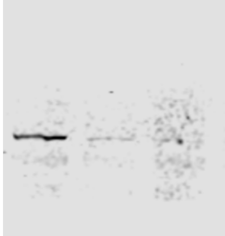
ACLY
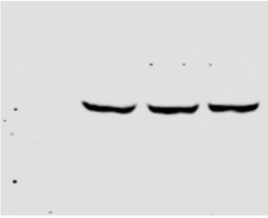
β-actin

Figure 4B

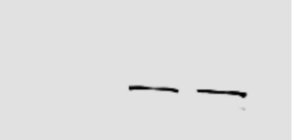
Flag-NAT10
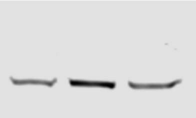
ACLY
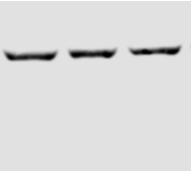
β-actin

Figure 4C

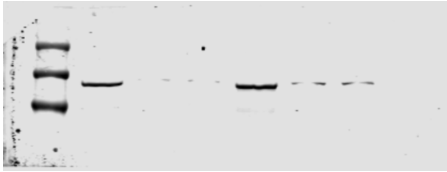
NAT10

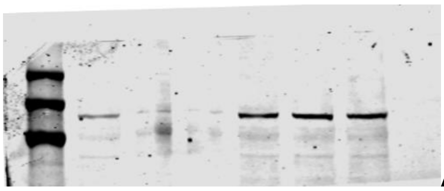
ACLY

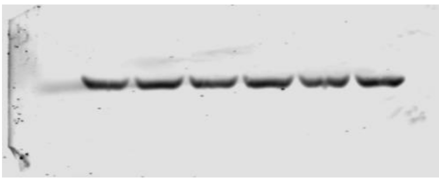
β-actin

Figure 4D

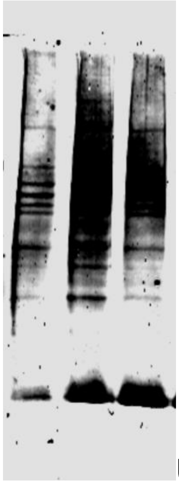
Ub-ACLY

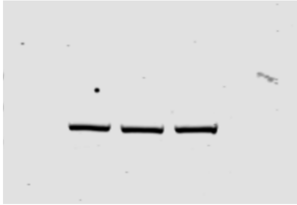
ACLY

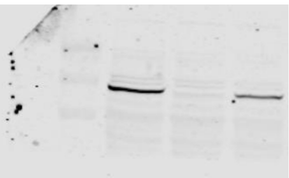
NAT10

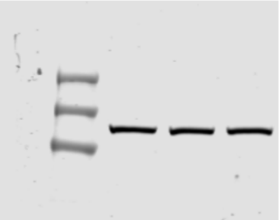
ACLY

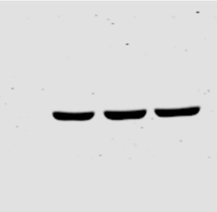
β-actin

Figure 4E

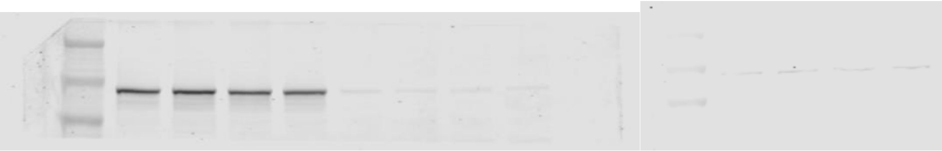
NAT10

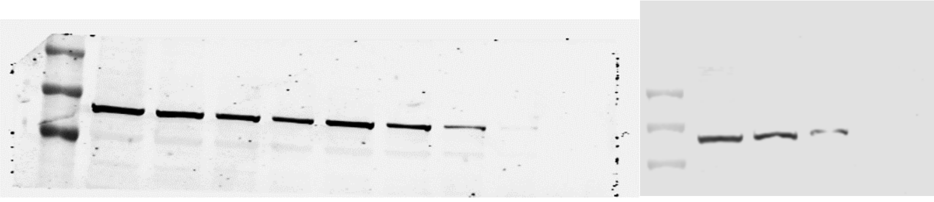
ACLY

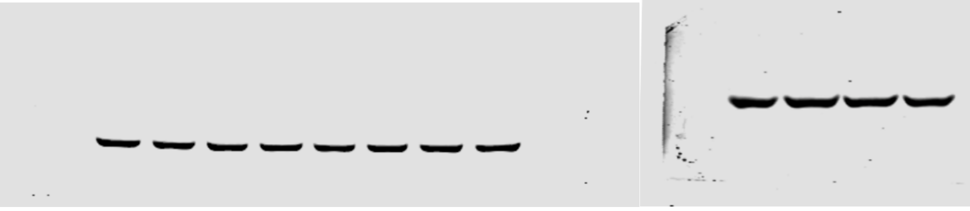
β-actin

Figure 4F

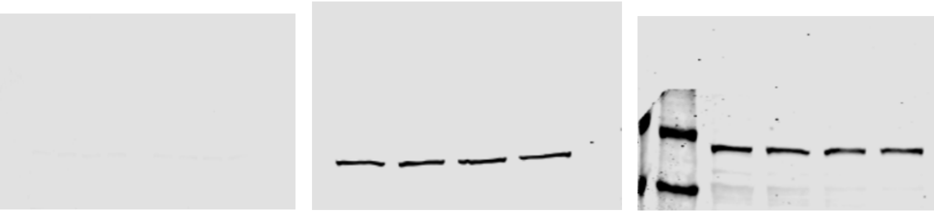

Flag-NAT10

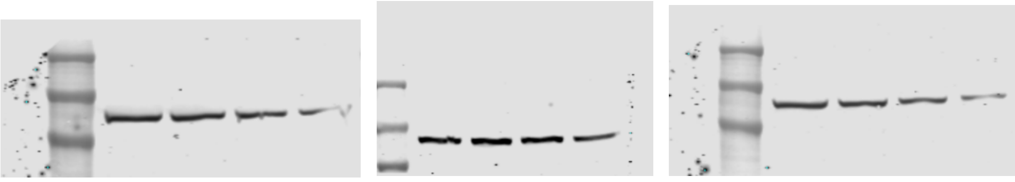

ACLY

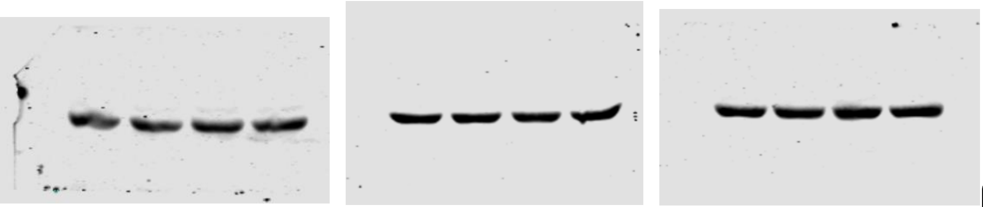

β-actin

Figure 5B

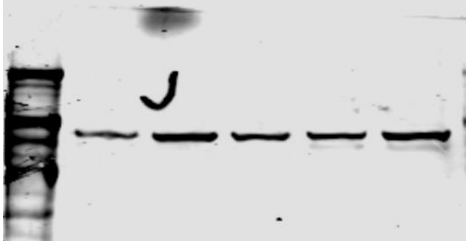

NAT10

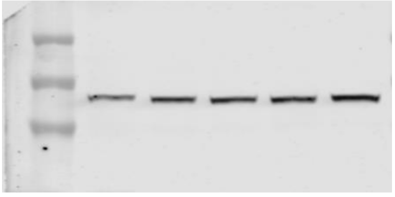

ACLY

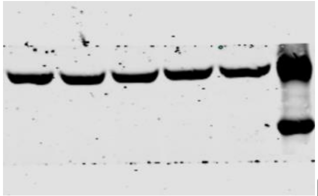

β-actin

Figure 5C

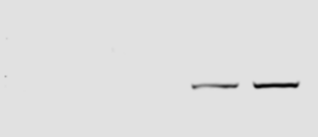

NAT10

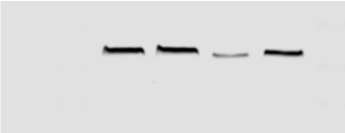

ACLY

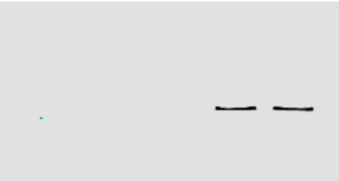

TOP1

α-tubulin

Figure5F

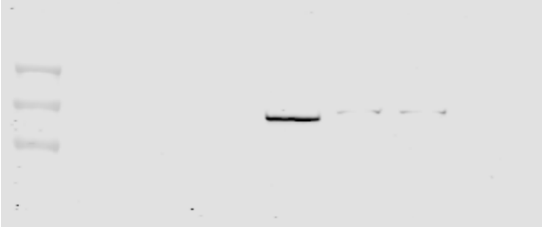
NAT10

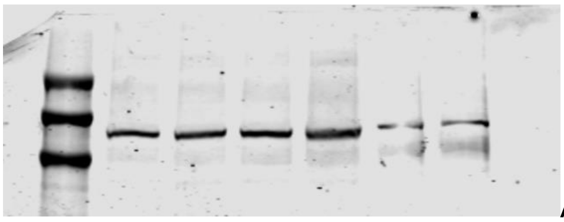
ACLY

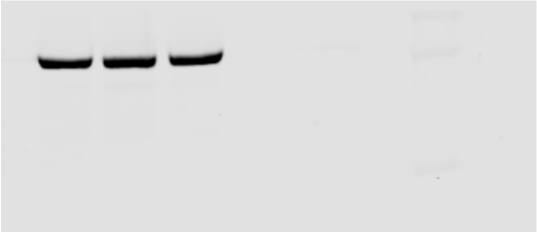
TOP1

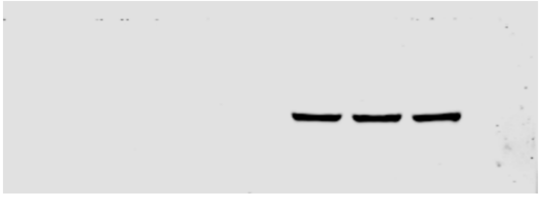
α-tubulin

Figure5G

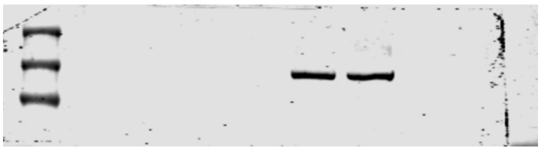
Flag-NAT10

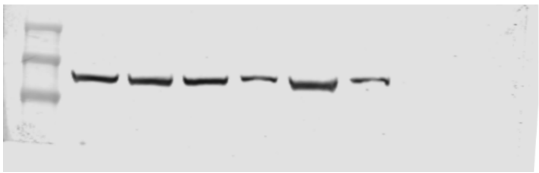
ACLY

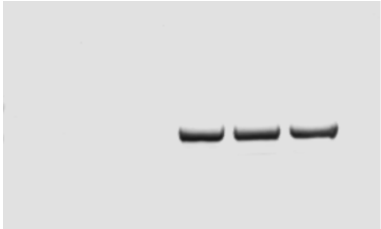
TOP1

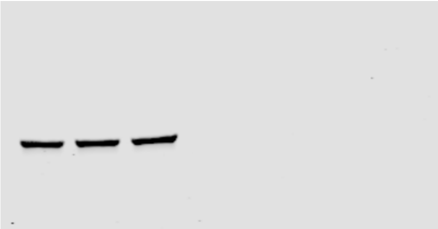
α-tubulin

Figure 6A

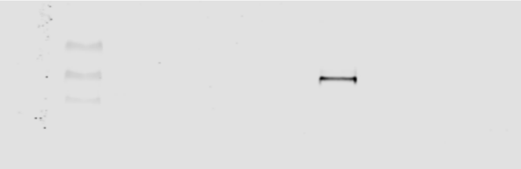
Ac-GST-ACLY

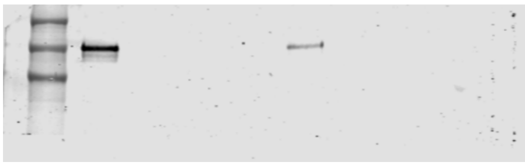
Ac-NAT10

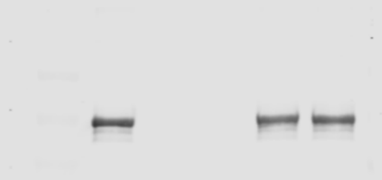
Flag-NAT10

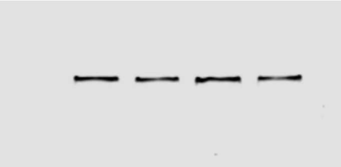
GST-ACLY

Figure 6B

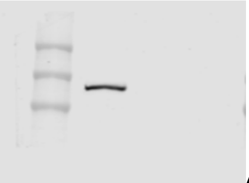
Ac-ACLY

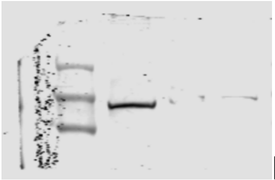
NAT10

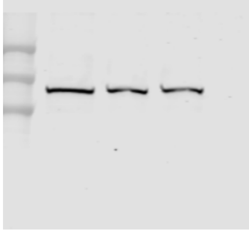
ACLY

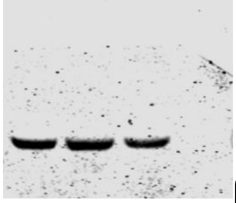
β-actin

Figure 6C

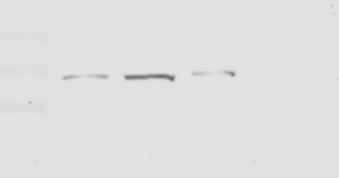
Ac-ACLY

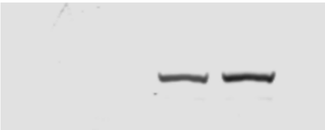
Flag-NAT10

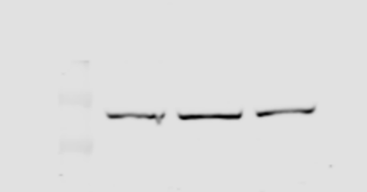
ACLY

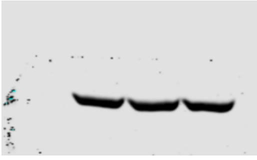
β-actin

Figure 6D

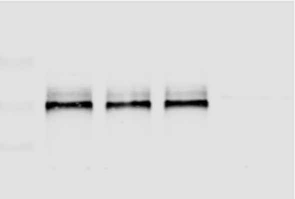
Flag-NAT10

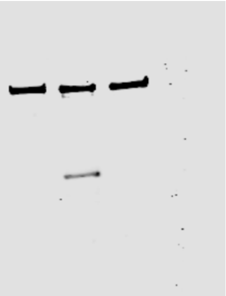
Ac-NAT10+ACLY-CCSα

Figure 6F

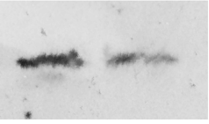
Ac-GST-ACLY

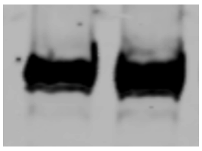
GST-ACLY

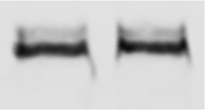
Flag-NAT10

Figure 6G

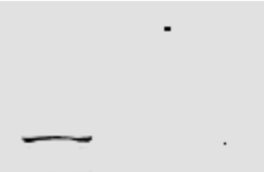
Ac-GFP-ACLY

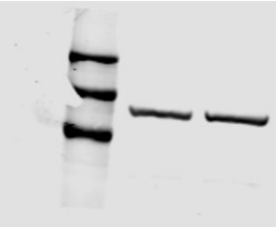
Flag-NAT10

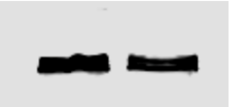
GFP-ACLY

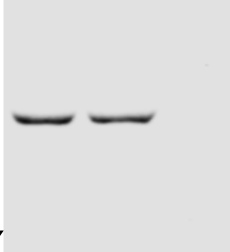
β-actin

Figure 6H

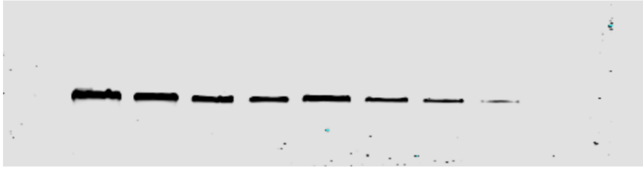
GFP-ACLY

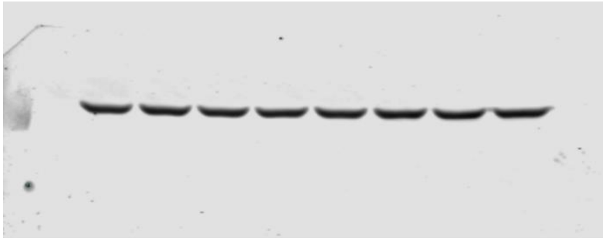
β-actin

Figure 6I

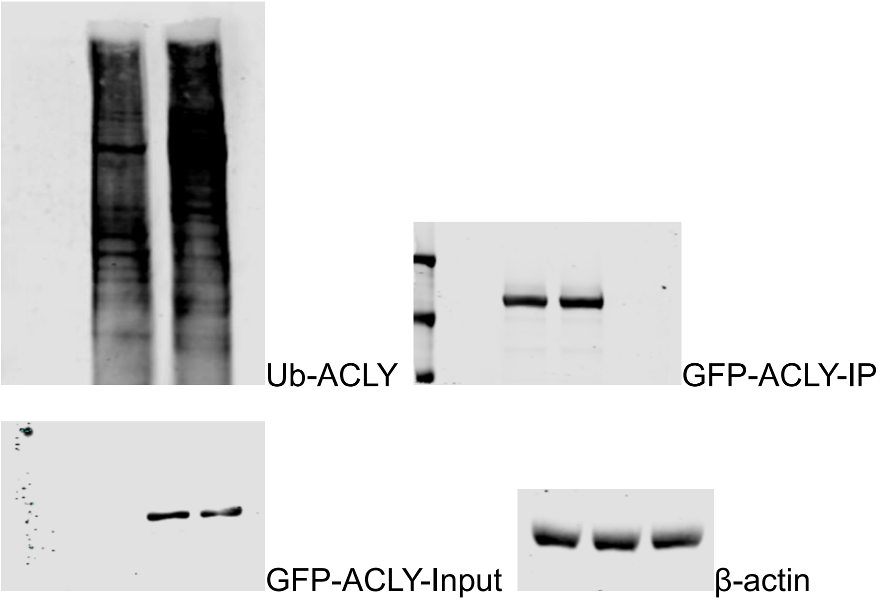

Figure 6K

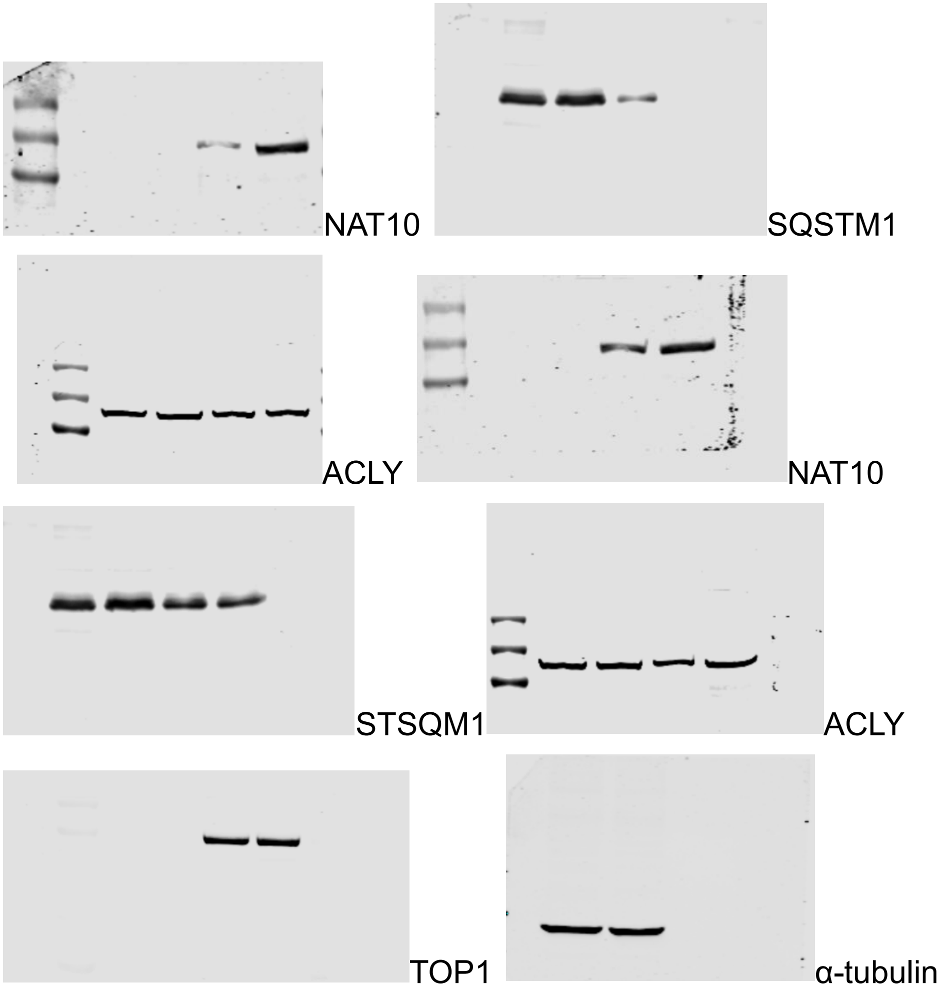

Figure 6L

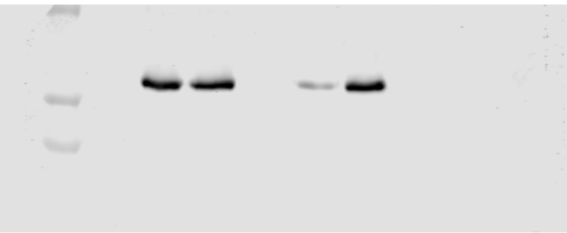

SQSTM1

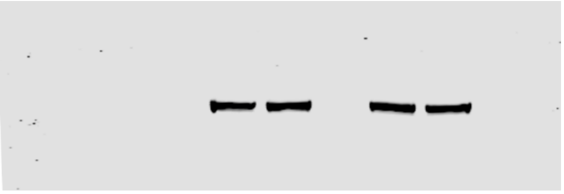

GFP-ACLY

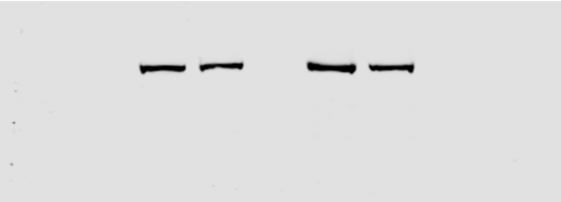

GFP-ACLY

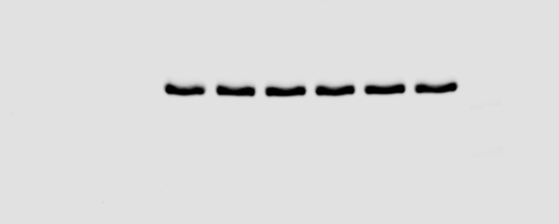

SQSTM1

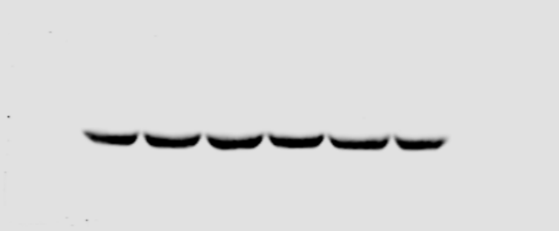

β-actin

Figure 6N

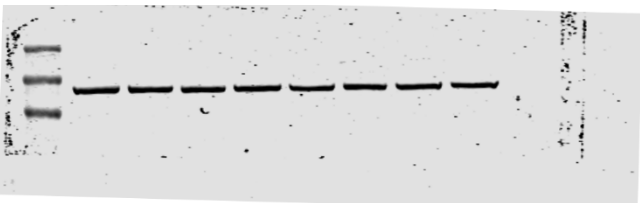

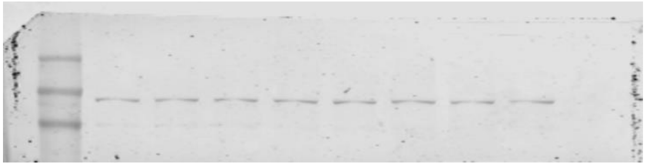

NAT10

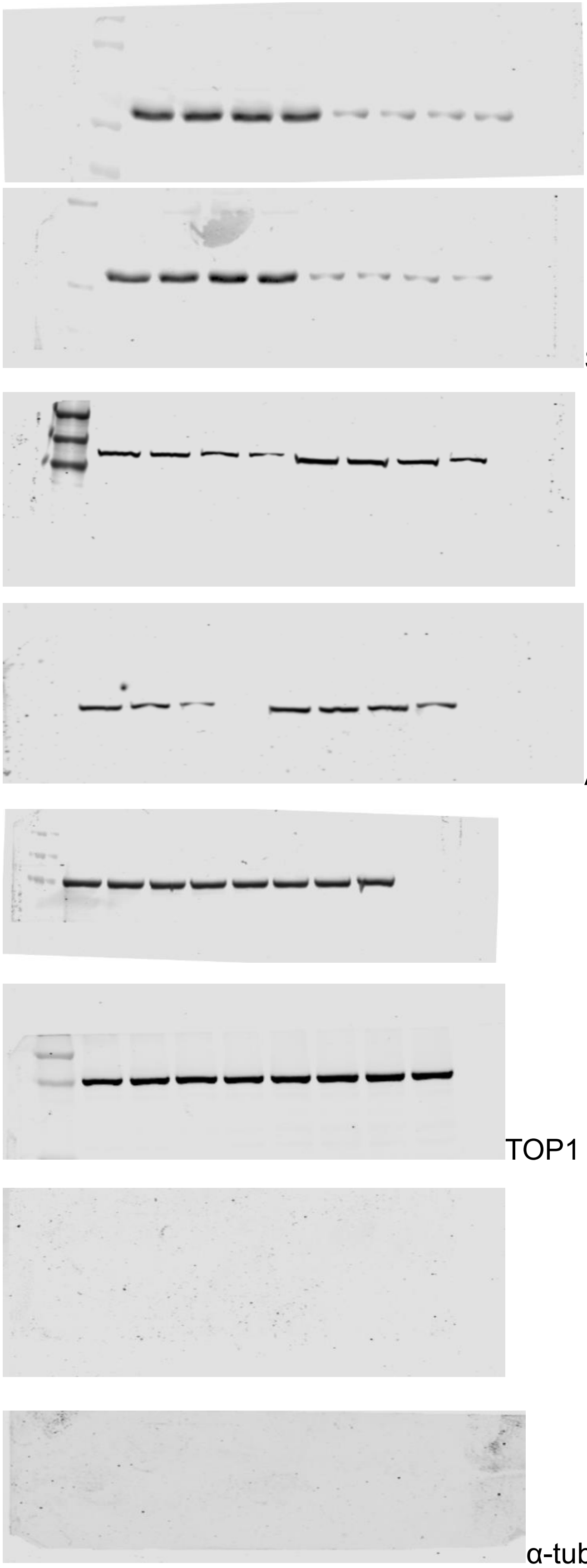

SQSTM1

ACLY

TOP1

α-tubulin

Figure 7B

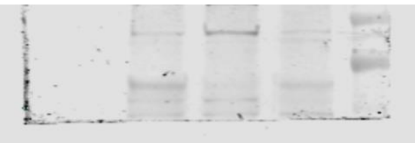

ACLY K468-Ac

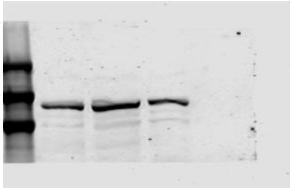

ACLY

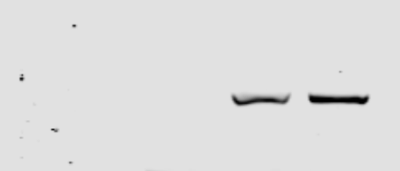

Flag-NAT10

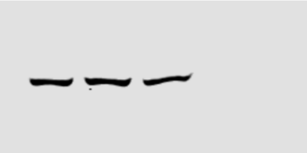

β-actin

Figure 7C

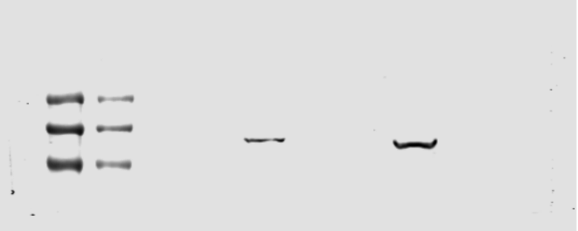

ACLY K468-Ac

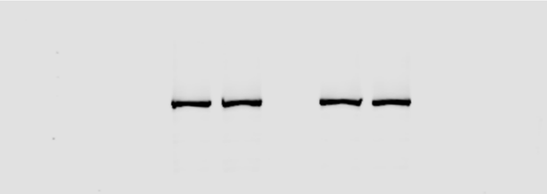

GFP-ACLY

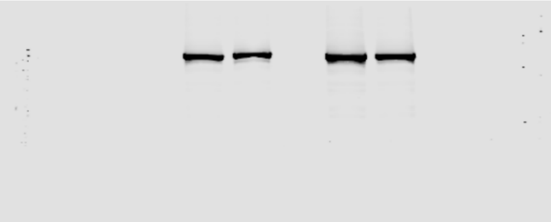

GFP-ACLY

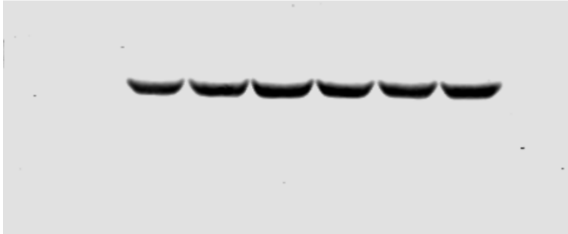

β-actin

Figure 7H

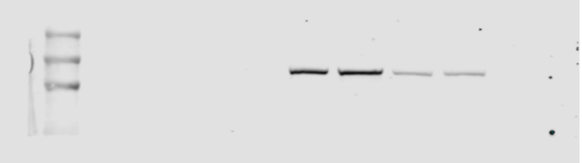

NAT10

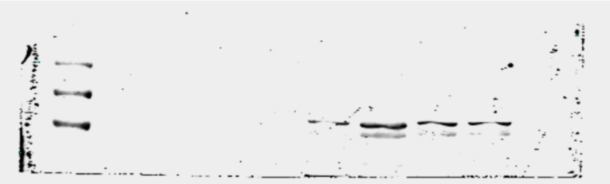
ACLY-K468-Ac

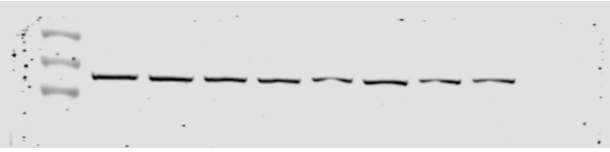
ACLY

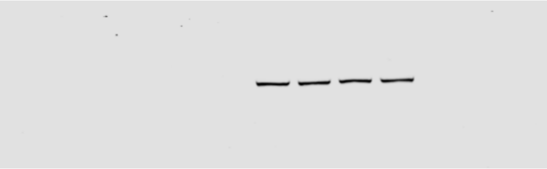
TOP1

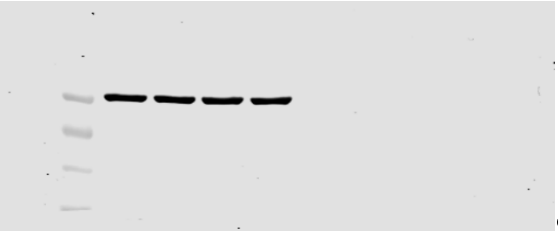
α-tubulin

Figure 8A

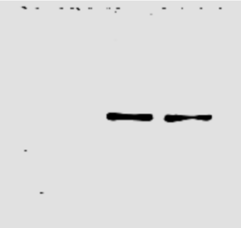
GFP-ACLY

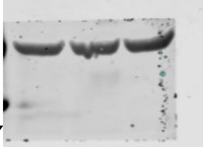
β-actin

Figure 8H

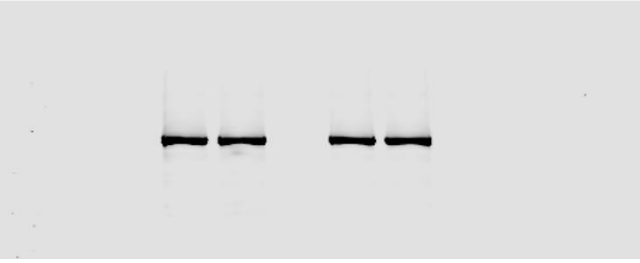

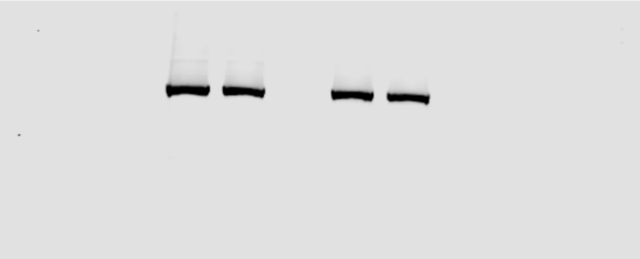

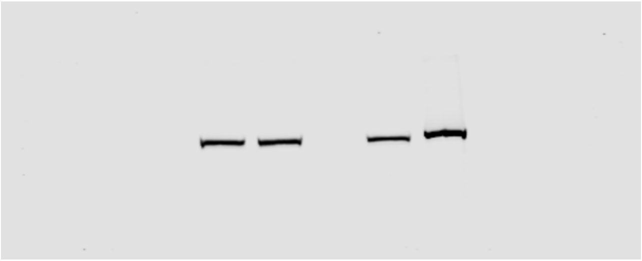

GFP-ACLY

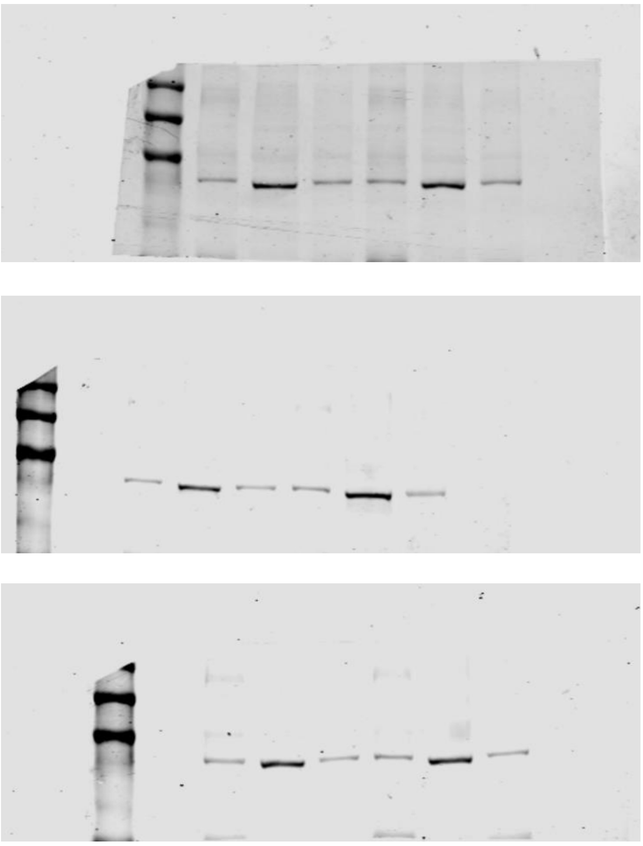

PIK3R1

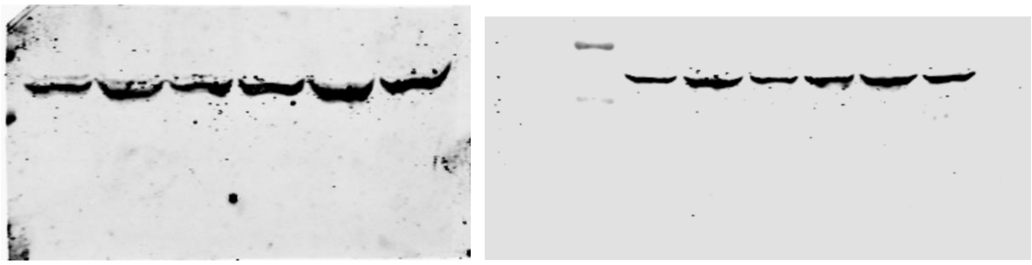

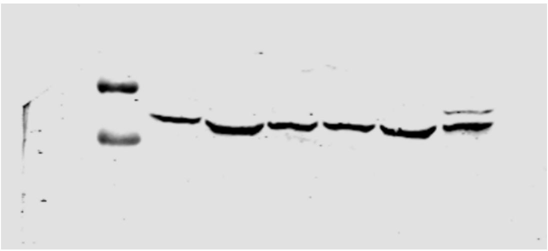

CYP2C9

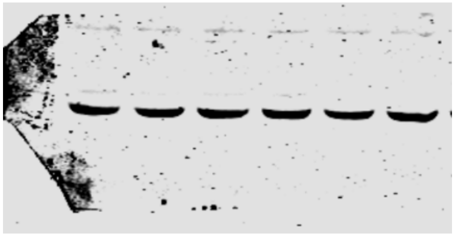

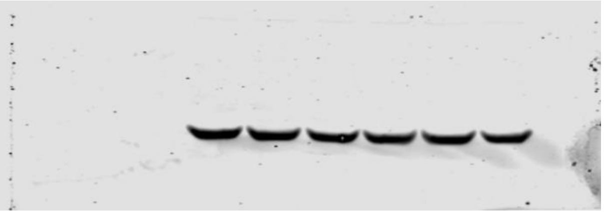

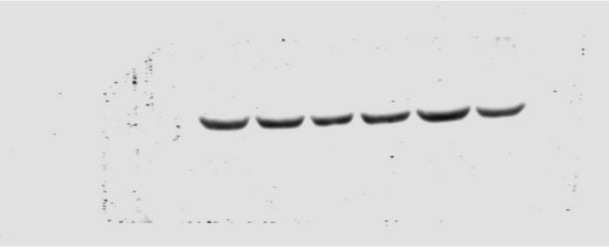

β-actin

Figure 8I

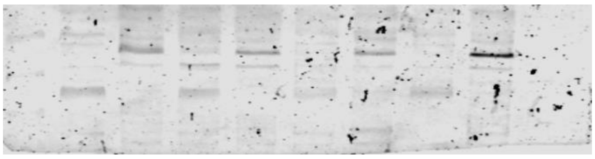

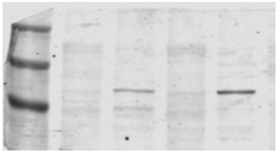

ACLYK468-Ac

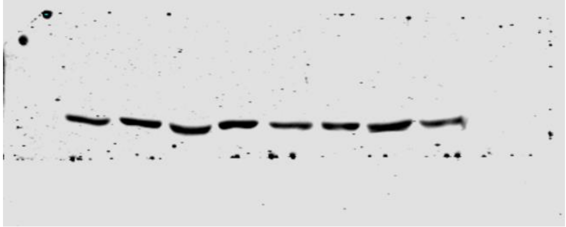

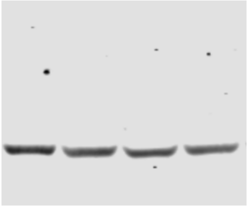

β-actin

Supplementary Figures

Figure2A

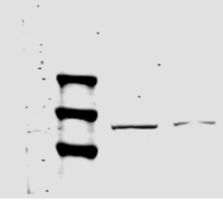

NAT10

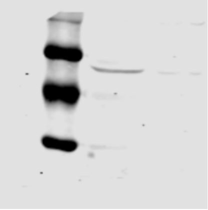

CYP2C9

β-actin

Figure 2B

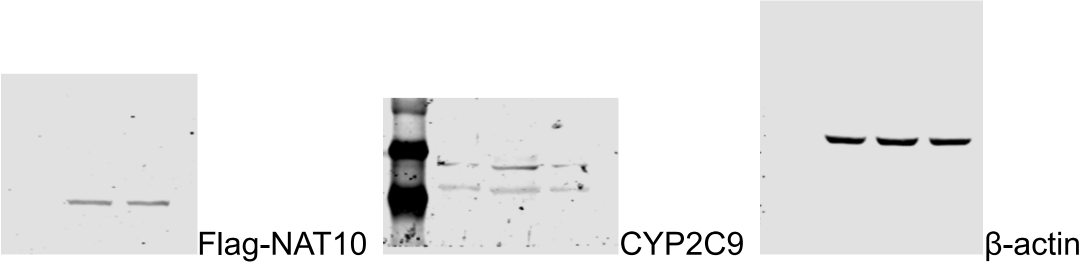

Figure 2C

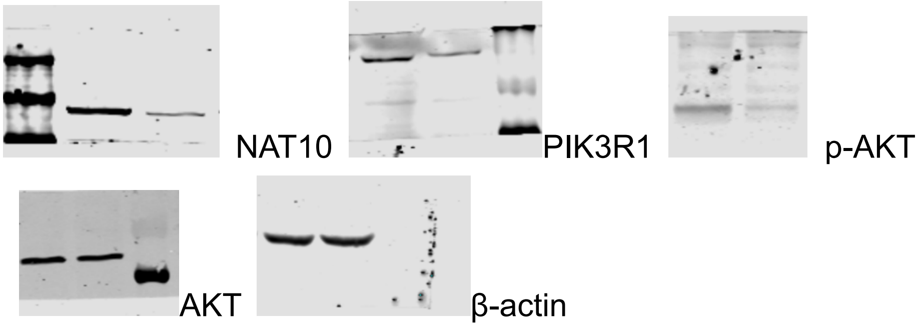

Figure 2D

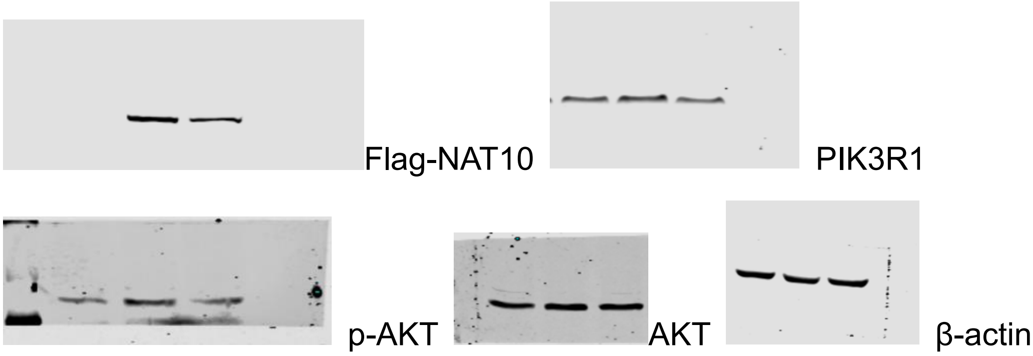

Figure 3B

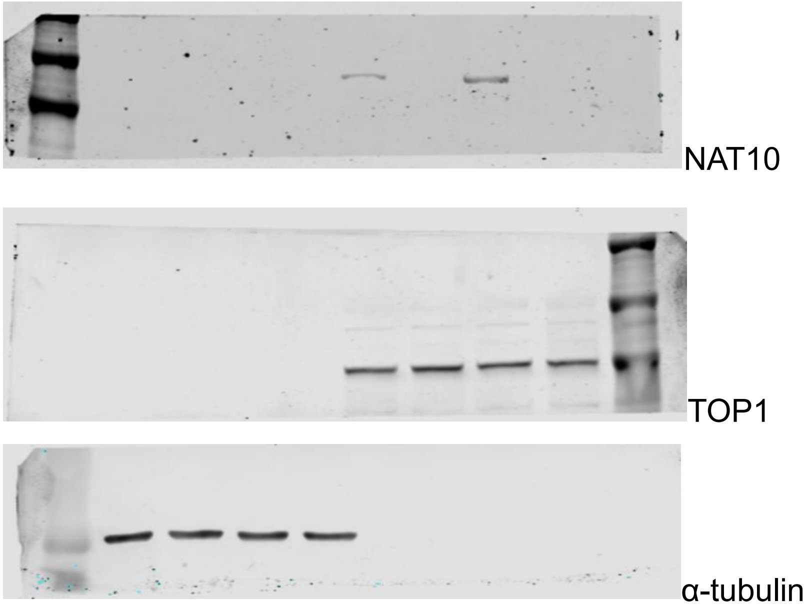

Figure 4A

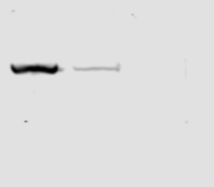
NAT10
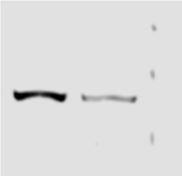
ACLY
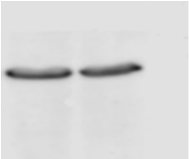
β-actin

Figure 4B

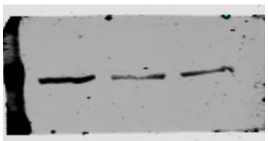
NAT10
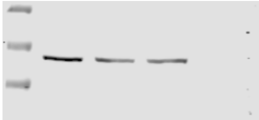
ACLY
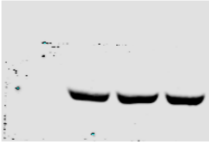
β-actin

Figure 5A

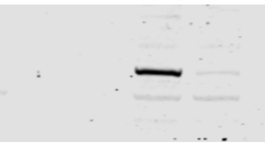
NAT10
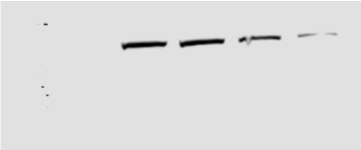
ACLY
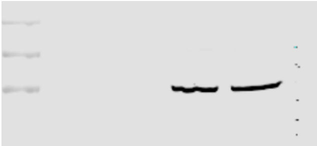
TOP1
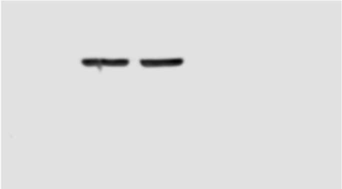
α-tubulin

Figure 5B

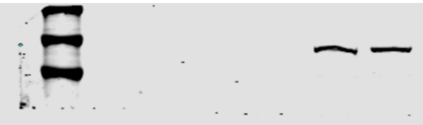
Flag-NAT10
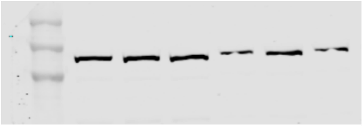
ACLY
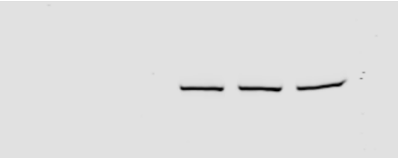
TOP1
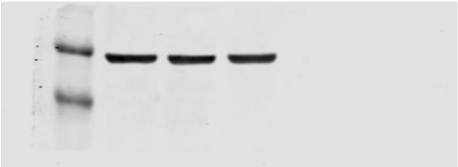
α-tubulin

Figure 6

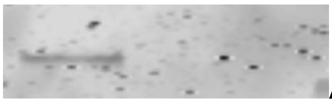
Ac-ACLY
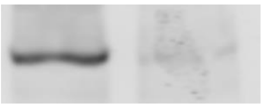
NAT10
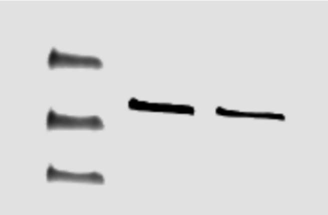
ACLY
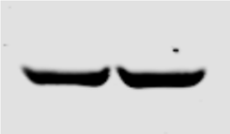
β-actin

Figure 8A

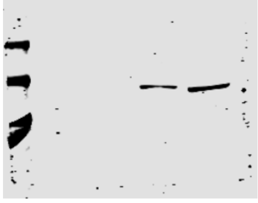
NAT10
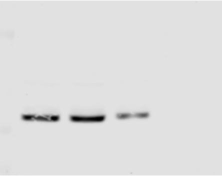
SQSTM1

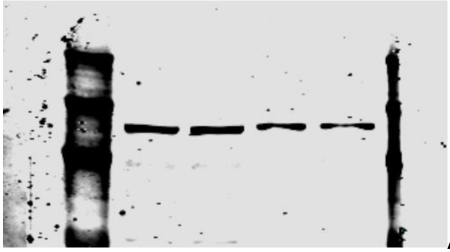
ACLY
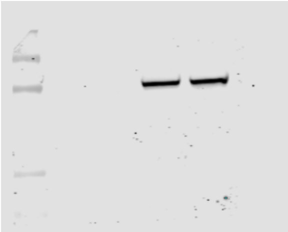
TOP1

α-tubulin

Figure 8B

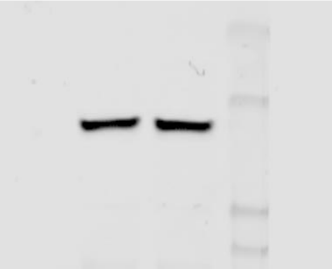
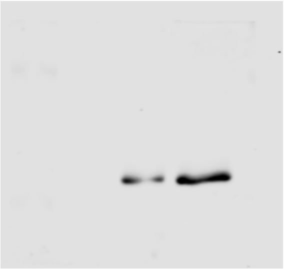
SQSTM1

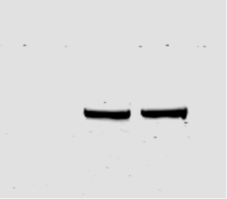
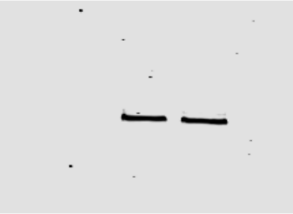
GFP-ACLY-IP

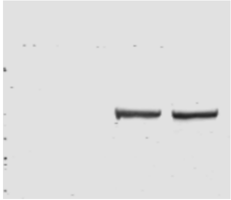
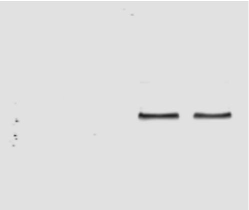
GFP-ACLY-Input

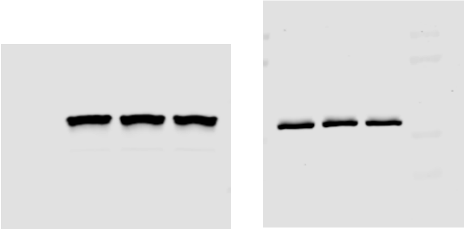

SQSTM1

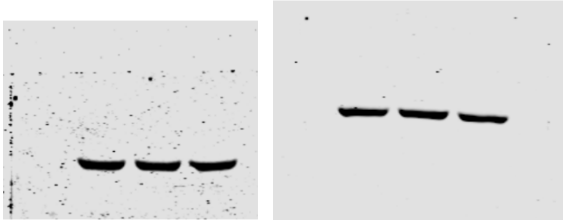

β-actin

Figure 9

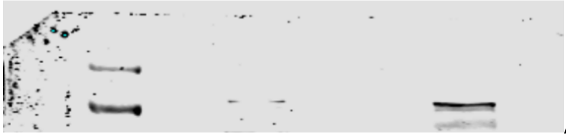

ACLY-K468-Ac

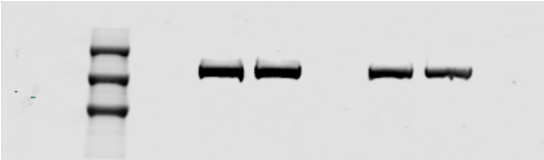

GFP-ACLY-IP

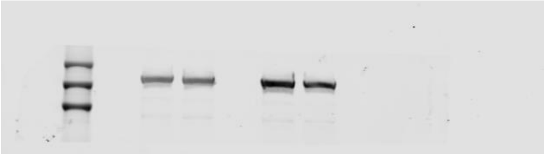

GFP-ACLY-Input

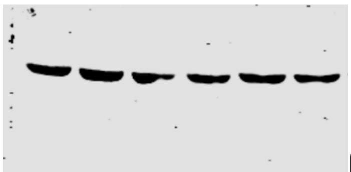

β-actin
